# Recent advances in microenvironment regulation for electrocatalysis

**DOI:** 10.1093/nsr/nwae315

**Published:** 2024-09-19

**Authors:** Zhiyuan Xu, Xin Tan, Chang Chen, Xingdong Wang, Rui Sui, Zhongbin Zhuang, Chao Zhang, Chen Chen

**Affiliations:** Engineering Research Center of Advanced Rare Earth Materials, Department of Chemistry, Tsinghua University, Beijing 100084, China; Engineering Research Center of Advanced Rare Earth Materials, Department of Chemistry, Tsinghua University, Beijing 100084, China; Engineering Research Center of Advanced Rare Earth Materials, Department of Chemistry, Tsinghua University, Beijing 100084, China; Research Institute of Petroleum Processing, SINOPEC, Beijing 100083, China; Engineering Research Center of Advanced Rare Earth Materials, Department of Chemistry, Tsinghua University, Beijing 100084, China; State Key Lab of Organic-Inorganic Composites, Beijing University of Chemical Technology, Beijing 100029, China; MOE International Joint Laboratory of Materials Microstructure, Institute for New Energy Materials and Low-Carbon Technologies, School of Materials Science and Engineering, Tianjin University of Technology, Tianjin 300384, China; Engineering Research Center of Advanced Rare Earth Materials, Department of Chemistry, Tsinghua University, Beijing 100084, China

**Keywords:** microenvironment regulation, electrical double layer, hydrogen electrocatalysis, oxygen electrocatalysis, CO_2_ reduction reaction

## Abstract

High-efficiency electrocatalysis could serve as the bridge that connects renewable energy technologies, hydrogen economy and carbon capture/utilization, promising a sustainable future for humankind. It is therefore of paramount significance to explore feasible strategies to modulate the relevant electrocatalytic reactions and optimize device performances so as to promote their large-scale practical applications. Microenvironment regulation at the catalytic interface has been demonstrated to be capable of effectively enhancing the reaction rates and improving the selectivities for specific products. In this review we summarize the latest advances in microenvironment regulation in typical electrocatalytic processes (including water electrolysis, hydrogen–oxygen fuel cells, and carbon dioxide reduction) and the related *in situ*/*operando* characterization techniques and theoretical simulation methods. At the end of this article, we present an outlook on development trends and possible future directions.

## Teaser

This review summarizes the latest advances in microenvironment regulation in typical electrocatalytic processes, including water electrolysis, hydrogen–oxygen fuel cells, and carbon dioxide reduction, and the related in situ/operando characterization techniques and theoretical simulation methods.

## INTRODUCTION

The pressing issue of continuously elevating CO_2_ concentrations in the Earth's atmosphere urgently calls for a fundamental paradigm shift in energy-related technologies and the recycling of this greenhouse gas, and electrocatalysis powered by sustainable energies is expected to play a substantial role in this grand picture [1]. Hydrogen economy and electrochemical CO_2_ reduction reaction (CO_2_RR) have been extensively acknowledged as two promising solutions. Green hydrogen produced via water electrolysis, in combination with hydrogen–oxygen fuel cells, could potentially lead to a sustainable energy infrastructure with net-zero carbon emissions [2]. On the other hand, CO_2_RR could recycle CO_2_ and convert it into value-added chemical feedstocks and fuels, representing an attractive alternative for closing the carbon cycle [3]. At the current stage, water electrolyzers, hydrogen–oxygen fuel cells, and CO_2_ electrolyzers have demonstrated considerable prospects for commercialization. However, there still exist quite a few obstacles on the way towards their large-scale application. For instance, water electrolyzers and hydrogen–oxygen fuel cells are facing the issues of sluggish kinetics of the oxygen reduction reaction (ORR) and oxygen evolution reaction (OER), unsatisfactory catalyst stability, and high costs due to inclusion of noble metal components [2,4,5]; for CO_2_RR, it is still rather challenging to achieve high selectivity for specific target high-value products [6]. Therefore, it is of great significance, both scientific and practical, to explore feasible strategies to boost the related electrocatalytic reactions.

Electrocatalytic reactions take place at the catalyst/electrolyte interface, where the electrical double layer (EDL) structure plays a crucial role in influencing reaction kinetics. Optimizing the catalytic structures and regulating the microenvironment at the interface are two effective options for promoting electrocatalytic processes [7]. The former aims to optimize the adsorption energies and activation barriers for the involved chemical species (including reactants, intermediates, and products) at the catalytically active sites in pursuit of high-performance catalysts, which has been one of the hottest topics in electrocatalysis during the past few decades [8]. Several review articles have already summarized research progress in the field of electrocatalysis, providing important guidance for the modulation of the microenvironment of electrocatalyst structures [9–11]. The latter, on the other hand, resorts to tactics such as controlling the electrolyte compositions (including pH, [12,13] anions [14–16] and cations [17,18]), altering the interfacial hydrophilicity/hydrophobicity [19–21], and constructing the organic-compound/electrode interface [22,23], and has also demonstrated its capabilities of elevating reaction rates, tuning reaction selectivities, and enhancing reaction stability. In addition, investigating the effect of microenvironment on the overall catalytic process could help to unveil the underlying catalytic mechanisms, and thus shed light on the development of next-generation electrocatalytic materials and devices [24]. For example, it has been reported that adding cyclohexanol could effectively block the direct adsorption of Nafion onto the Pt surface [25], and that employing chemically modified carbon supports with tailored porosity enabled the uniform dispersion of ionomer [26]. These strategies have successfully optimized the interface microenvironment of ionomers/catalysts, thereby improving the operational performance and robustness of fuel cell devices. In addition, modulating the cation concentrations [17], local pH [27] and local CO_2_/H_2_O ratio [28] at the catalytic interface could enable production of multi-carbon products via CO_2_RR. These pioneering works showcase the importance of microenvironment regulation for promoting practical applications. On the other hand, there are still some issues that need to be noted: most of the research works on microenvironment were based on the three-electrode system rather than practical devices. Similar to the challenges faced in catalyst development, a number of extraordinary results have been obtained using the rotating disk electrode (RDE) setup, and yet they could hardly be reproduced in the membrane electrode assembly (MEA) configuration [29]. This is probably because when the catalytic reactions are transferred from the planar electrodes in the conventional three-electrode setup to the triple-phase interface in MEA, the distinct differences in their operational conditions and microenvironments could lead to significant discrepancies [30]. Therefore, it is necessary to conduct cross validation between the three-electrode system and MEA for the research on microenvironment in the future. To sum up, the approach of starting from the electrode/electrolyte interface could help to establish a more comprehensive and in-depth understanding on the fundamentals of electrocatalysis, to explore feasible strategies for regulating the microenvironment, and to develop advanced electrochemical devices with superior performances.

**Figure 1.**
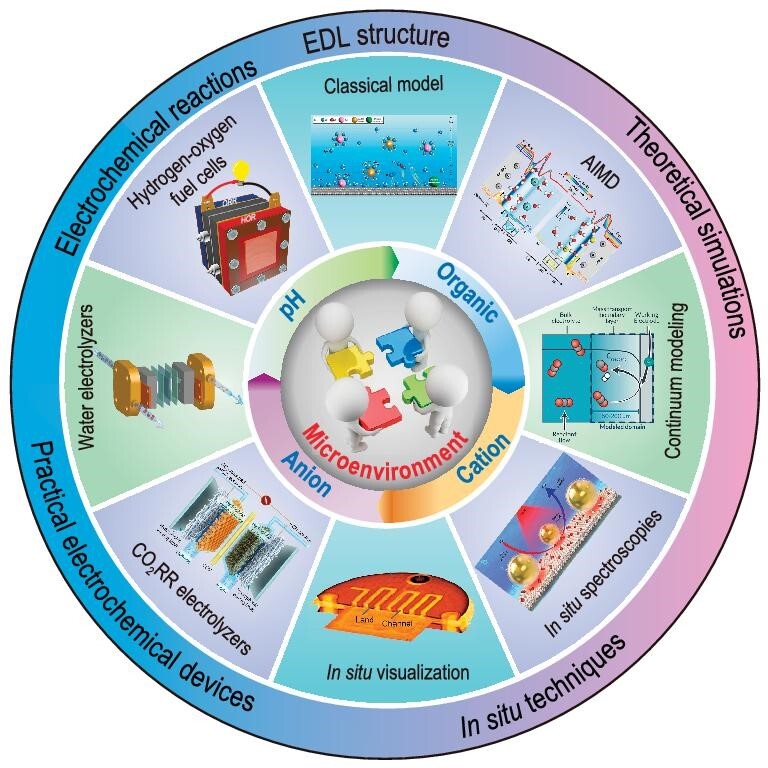
Schematic illustration of microenvironment regulation for electrocatalysis.

Here in this paper, we present a brief review on the recent advances in microenvironment regulation for typical energy-related electrochemical processes (such as HOR, HER, ORR, OER, CO_2_RR) associated with water electrolyzers, hydrogen–fuel cells, and CO_2_ electrolyzers (Fig. 1). In the following sections, we first give an introduction on the establishment and development of EDL theory. Then we overview the latest advances in the research on microenvironment regulation, with particular emphasis on the effects of pH, anions, cations and organic compounds, and on the underlying principles for different regulation strategies. Next we summarize the most recent progress in the design and fabrication of membrane electrodes and corresponding electrocatalytic systems based on microenvironment regulation. An extra section is given for the *in situ*/*operando* characterization techniques (such as *in situ* visualization, surface-enhanced infrared/Raman spectroscopies) and theoretical simulation methods (such as *ab initio* molecular dynamics and continuum modeling). At the end of this review, we present a brief summary on the challenges currently facing this research area, and an outlook on possible future directions. We believe this comprehensive review of recent advancements in microenvironment regulation across a broad spectrum of electrochemical processes, coupled with practical insights into device-level optimization and *in situ*/*operando* characterization, can help to deepen understanding and facilitate the advance in microenvironment engineering.

## ELECTRODE–ELECTROLYTE INTERFACES: ELECTRICAL DOUBLE LAYER (EDL) STRUCTURE

**Figure 2.**
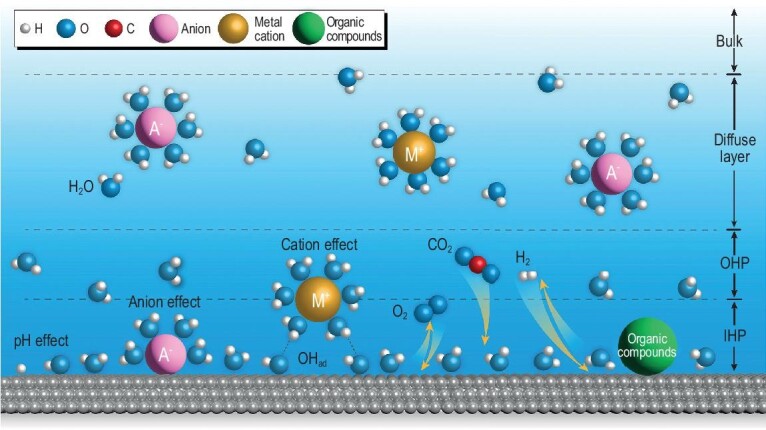
Schematic illustration of the EDL structure and the typical effects of microenvironment in electrocatalysis. The off-white, blue, red and silver-white spheres represent the H, O, C and the metal (electrode material) atoms, respectively. The golden, pink and green spheres represent the cations, the anions and organic species, respectively.

The microenvironment of electrode–electrolyte interface (EEI) could have a significant impact on the thermodynamics, kinetics, reaction pathways and thus product selectivity. On the other hand, the electrocatalytic reactions themselves could also alter the interfacial microenvironment, such as the change of electric field, dynamic adsorption/desorption of reactants, intermediates and products at the electrode surface; in particular, when the reaction involves the generation/consumption of H^+^/OH^−^, the local pH at the electrode surface could deviate substantially from the bulk electrolyte. Understanding the structure of EEI at the atomic/molecular scale is a major challenge owing to the dynamic and complex interactions between electrocatalytic reactions and the interfacial environment [31]. Electrical double layer (EDL) theory lays the foundation of theoretical research on the EEI, and during nearly two centuries of development, the EDL model has undergone significant modifications, currently leading to the well-known Gouy–Chapman–Stern (GCS) model. After further incorporating the solvated ions and adsorption [32], the schematic illustration of the classic EDL model is shown in Fig. 2, which has been widely applied in the field of electrocatalysis.

The EDL extends from the electrode towards the electrolyte, with the layer nearest to the electrode surface composed of solvent molecule dipoles arranged in an ordered manner, forming the inner Helmholtz plane (IHP). Certain specific adsorbed anions may lose a portion of their solvation shells and directly adhere to the electrode surface, which might compete with the adsorption of the reactant/intermediate species. On top is the outer Helmholtz plane (OHP) formed through the solvation of cations. On account of the high solvation enthalpies of cations, it is generally believed that cations are not in direct contact with the electrode. Nonetheless, the electrode interface would still be affected by the quasi-specific adsorption of cations coupling with other surface-adsorbed species (such as *OH) [18]. The electric potential in the vicinity of the electrode interface region (including IHP and OHP) is associated with the surface charge of the electrode, and follows a quasi-linear distribution. Farther out from OHP is the diffuse layer, where the distribution of cations/anions in this layer is still controlled partially by the electric potential, which varies almost exponentially with respect to the distance from the electrode.

The classic EDL model described above is the foundation for understanding the interfacial microenvironment, but it still has significant limitations. For example, the structure of interfacial water molecules, the degree of ion hydration and the position of cations at the interface are still rather vague. Recently, Bonn *et al*. discussed the formation of EDL on different types of charged interfaces and its impact on the interfacial water structure, emphasizing the limitations of the classic mean-field description of EDL in depicting the real systems involving charged surfaces, water, and ions at the molecular level [33]. Taking into consideration more microscopic details (such as interfacial water properties and specific ion interactions) as well as developing density functional theory (DFT)-based molecular dynamics simulations has positive implications for understanding interfacial water structures. *In situ* spectroscopies are crucial for probing the microscopic details of interfacial water structures. Tian *et al*. observed the interfacial water structure on electrified Au single-crystal electrode surfaces via *in situ* Raman spectroscopy [34]. They discovered that the O–H stretching vibrational mode of interfacial water underwent two transitions, accompanied by two changes in the number of hydrogen bonds when sweeping to negative potentials. In combination with *ab initio* molecular dynamics (AIMD) simulations, it was demonstrated that these transitions are due to the evolution of interfacial water molecules from the ‘parallel’ configuration to ‘one-H-down’ and then to ‘two-H-down’ configurations with the variation in interfacial electric potential.

Furthermore, the determination of actual parameters for the EDL, such as the potential drop (PD) and the potential of zero charge (PZC), is crucial for validating models and making necessary adjustments. Crumlin *et al*. directly probed the electric potential distribution in the EDL at the interface between electrified gold polycrystalline electrode and liquid electrolyte under polarization conditions via ambient-pressure X-ray photoelectron spectroscopy [35]. This not only discerned the shape of the EDL profile, but also helped to further understand the influence of electrolyte concentration and applied potential on the EDL structure. Koper *et al*. determined the PZC as 0.3 V *vs*. normal hydrogen electrode (NHE) on Pt(111) by measuring the Gouy–Chapman capacitance minimum at the interface between Pt(111) and aqueous perchlorate electrolyte [36]. Owing to the stronger ‘effective screening’ than predicted by purely electrostatic mean-field Poisson–Boltzmann theory, the actual diffuse double layer structure of Pt(111) deviates more from the theoretical values. Recently, Suntivich *et al*. reported a novel optical method for determining the PZC, which directly probes the electric field at the electrochemical interface through field-induced second-harmonic generation (SHG) effects [37]. Using this method, the team determined the PZC value of the polycrystalline Pt/water interface to be 231 ± 76 mV *vs*. standard hydrogen electrode (SHE). This phase-sensitive SHG method, which does not require probe molecules, also provides a promising approach to determining the PZC of other metal electrodes under different electrochemical conditions.

With the advance of *in situ* testing techniques and theoretical simulation methods, new impetus has been added to the development of EDL models. Understanding of the EDL structure at the atomic scale is of important significance for better utilizing the microenvironment to regulate electrocatalytic performances.

## MICROENVIRONMENTAL REGULATION FOR HYDROGEN ELECTROCATALYSIS

**Figure 3.**
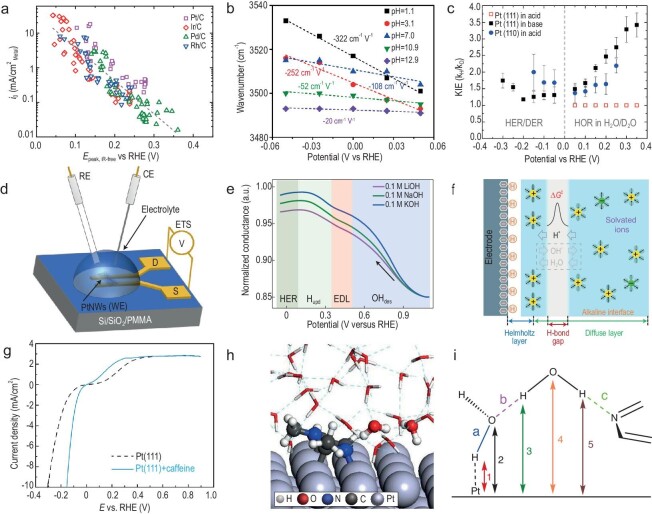
Microenvironmental regulation for hydrogen electrocatalysis. (a) The exchange current density of HOR/HER on Pt/C, Ir/C, Pd/C and Rh/C as a function of *E*_peak_. Reproduced with permission from Ref. [12]. Copyright 2016 American Association for the Advancement of Science. (b) The O–H stretching wavenumber of interfacial water as a function of potential. Reproduced with permission from Ref. [43]. Copyright 2020 American Chemical Society. (c) KIE of HER/HOR on Pt(111) and Pt(110) in HClO_4_/DClO_4_ and KOH/KOD. Reproduced with permission from Ref. [44]. Copyright 2020 American Chemical Society. (d) Schematic of operando ETS measurements and (e) the normalized ETS conductance of the PtNW device in 0.1 M MOH (M = Li, Na and K) electrolyte solutions as a function of potential. Reproduced with permission from Ref. [47]. Copyright 2022 Nature Publishing Group. (f) Schematic diagram of the proton transfer from diffuse layer to Helmholtz layer across the H-bond gap in the alkaline EDL. Reproduced with permission from Ref. [48]. Copyright 2022 Nature Publishing Group. (g) HER/HOR polarization curves of Pt(111) and Pt(111) + caffeine in 0.1 M KOH. Reproduced with permission from Ref. [49]. Copyright 2020 American Chemical Society. (h) Atomic configuration of an equilibrated H_2_O–Me-N_1_C_2_–Pt(100) interface. The grey, brown, blue, red and white spheres represent Pt, C, N, O and H atoms, respectively. (i) Schematic illustration of an interfacial water dimer on Pt(100) interface with Me-N_1_C_2_. (h, i) Reproduced with permission from Ref. [22]. Copyright 2023 Nature Publishing Group.

Electrochemical hydrogen evolution and oxidation reactions (HER/HOR) are not only the foundation of a sustainable hydrogen economy, but also the typical processes for electrocatalytic reactions, serving as models for studying electrode kinetics and the fundamental principles of electrocatalysis. The non-Nernstian pH dependency of the HER/HOR kinetics leads to notably lower reaction rates in alkaline environments than under acidic conditions, and the origins are still unclear [38]. Yan *et al*. assessed the HER/HOR activities of Pt catalysts and the oxidation peak for the underpotentially deposited hydrogen (H_upd_) in different buffer solutions over a wide pH range (0–13). It was found that as the pH increases, the HER/HOR activities gradually decrease whereas the H_upd_ peak potential (*E*_peak_) shifts anodically [39]. Nørskov *et al*. established a direct correlation between *E*_peak_ and the hydrogen binding energy (HBE) of Pt based on DFT calculations [40], and thus the pH effect on *E*_peak_ seems to suggest that HBE is the sole descriptor of the HER/HOR activities on Pt. As is shown in Fig. 3a, a universal dependence between pH and the measured HBE has also been found on Pt-group metals other than Pt (including Ir, Pd and Rh) [12]. This provides an explanation for the suppressed HER/HOR activities in alkaline solutions, that the OH species would lead to the overbinding of H_ad_ owing to the higher HBE.

However, it does not seem sufficiently rigorous to directly associate the measured H_upd_ peak with HBE. The similar pH dependence of H_upd_ peaks on different metals suggests that this is not related to the innate properties of electrodes, but rather dominated by the interfacial microenvironment. Therefore, the apparent HBE (HBE_app_), which takes the Gibbs free energy of interfacial water adsorption (WBE) into consideration, was proposed as a more proper descriptor for hydrogen electrocatalysis. Koper *et al*. suggested that the non-Nernstian pH shift of H_upd_ peak is due to the fact that the co-adsorbed alkali metal cations along the step weaken the OH adsorption at the step sites on Pt(553) [41]. Xu and Yan *et al*. combined cyclic voltammetry and surface-enhanced infrared absorption spectroscopy (SEIRAS), and proposed that the pH-dependent shift of the H_upd_ peak on Pt may be attributed to the altered structure of interfacial water by the electrode potential, rather than by cations [42]. Shao *et al*. tracked the changes in HBE and WBE on Pt thin film under different pH conditions, and claimed that the experimentally determined HBE is actually the combined result of electric field, H_ad_ coverage, Pt–water and H_ad_–water interactions [43]. In electrolytes with higher pH values, the lowered WBE leads to an increased distance between the Pt surface and the interfacial water layer, thus hindering charge transfer and resulting in slower reaction kinetics (Fig. 3b). The results on the kinetic isotope effect (KIE) of HER/HOR on single-crystalline Pt(111) and Pt(110) also support the significant influence of pH on water structure at the interface [44]. No significant KIE for hydrogen reactions was observed in acidic media, but a KIE as high as 3.4 was observed for HOR on Pt(111) in alkaline solution, indicating that interfacial water plays a crucial role in promoting proton adsorption and overall hydrogen reaction kinetics in alkaline media (Fig. 3c). The interfacial electric field is also considered an intrinsic origin of the pH effect on hydrogen electrocatalysis. Surendranath *et al*. quantified the interfacial electric field strength on Pt as a function of pH by using a non-Faradaic reaction probe and tracking the pH sensitivity of H_2_/H^+^ catalysis [45]. The results show that with each unit increment in pH, the electrostatic potential at the Pt surface increases significantly, indicating a distinct difference in electric field strengths between acidic and alkaline conditions. The pronounced difference in interfacial electric field strength at RHE would influence the rate of solvent molecule reorganization, thereby contributing to the pH dependence of HER/HOR kinetics.

Different alkali metal cations (AM^+^) have been found to significantly affect the HER in alkaline solutions, but the cation effect is rather insignificant in acidic media. This may be due to the presence of a large amount of OH species in alkaline solutions, leading to a more complex interfacial microenvironment, with the cations believed to influence the interfacial solvent layer through these OH_ad_ species. Jia *et al*. investigated the cation effect on hydrogen electrocatalysis in Pt, Ni and PtNi systems, and found that Li^+^ does not affect the HER on Pt, but significantly improves the HER on Ni and PtNi [46]. This implies that Li^+^ could not only facilitate the OH_ad_ removal by forming a OH_ad_–(H_2_O)*_x_*–Li^+^ structure, but also hinders HOR by weakening the adsorption strength of OH_ad_. It was also observed that increasing the Li^+^ concentration only promotes HER, whereas changing the identity of AM^+^ affects both HER/HOR on Pt. These experimental results collectively indicate that the cation effect could alter the rate of hydrogen electrocatalytic reactions by influencing the OH_ad_ on Pt. Duan *et al*. investigated the cation effects of HER on Pt via electrochemical impedance spectroscopy and electrical transport spectroscopy (Fig. 3d, e) [47]. *In situ* experimental evidence on surface adsorbates and charge-transfer resistance (*R*_ct_) at the electrode/electrolyte interface indicates that the high polarity of OH_ad_ allows it to act as electrically favored proton acceptors and geometrically favored proton donors, thereby accelerating the kinetics of the Volmer step. Cations with smaller atomic numbers (such as Li^+^) could minimize the interference with OH_ad_ on Pt, resulting in a higher OH_ad_ coverage and thus a better HER activity than Na^+^ and K^+^. These works imply that the cation effect is often closely related to the OH_ad_. Although the bifunctional mechanism of hydrogen electrocatalysis in alkaline is still a topic of much debate, it is almost unanimously agreed that OH_ad_ has a significant impact on the kinetics of HER/HOR. Chen *et al*. conducted *in situ* surface-enhanced infrared absorption spectroscopy and AIMD simulations, and argued that the main origin for the suppressed activity of HER/HOR on Pt in alkaline electrolytes is the presence of water gaps in the EDL and the weakened connectivity of hydrogen bond networks (Fig. 3f) [48]. Furthermore, OH_ad_ could significantly improve the connectivity of the hydrogen bond network, which explains the fact that the hydrogen reaction activity of PtRu is higher than that of Pt in alkaline media.

In addition to electrolytes, some recent studies have shown that organic additives adsorbed on electrode surfaces can fine-tune the interfacial microenvironment and improve the kinetics of alkaline hydrogen reactions. Snyder *et al*. used caffeine adsorbed on Pt surface as a ‘double-layer dopant’ and enhanced the rates of HER/HOR on Pt by more than 5-fold (Fig. 3g) [49]. Fourier Transform Infrared Spectrometer (FTIR) spectroscopy provides evidence that the caffeine molecules are adsorbed on the Pt surface and can be stabilized and regenerated through electrochemical deposition during load/potential cycling. The hydrophobic and oxophobic nature of caffeine can inhibit electrode corrosion and thus enhance catalytic stability. In addition, its slight polarity weakens the OH_ad_, leading the team to speculate that it may affect the hydrogen bonding network and the mobility of surrounding water molecules, thus leading to an elevated rate of hydrogen reactions. Inspired by the intriguing results regarding caffeine, Xu *et al*. used theophylline derivatives with partial structural similarities to caffeine and investigated the effect of different molecular fragments of caffeine on the HOR/HER activity on Pt in alkaline media [50]. *In situ* Raman spectroscopy provided the intensity of weakly hydrogen-bonded water on Pt decorated with 7-alkyl substituted theophylline derivatives of varying alkyl chain lengths, consistent with the trend of corresponding HOR/HER exchange current densities. This indicates that theophylline derivatives can enhance the HOR/HER activity by promoting interface water reorganization through increased weakly hydrogen-bonded water. In addition to caffeine-like reagents, Jia *et al*. found that *N*-methylimidazoles at the Pt–water interface can also accelerate the hydrogen reaction [22]. The more negatively charged pyridinic nitrogen (N_3_) in *N*-methylimidazole forms a strong N_3_···H_2_O bond with interfacial H_2_O, keeping the second layer of water molecules close to the Pt surface to facilitate OH^−^ diffusion at the interface, thereby accelerating the Volmer step on Pt (Fig. 3h, i). This organic interface strategy has been shown to be quite feasible for practical devices, with a 40% performance enhancement achieved in an anion exchange membrane electrolyzer by adding 1,2-dimethylimidazole to the Pt cathode.

As manifested in the non-Nernstian pH effect, the interface microenvironment could have profound influences on the HER/HOR kinetics. Current research suggests that the underlying mechanisms may involve alteration in the EDL structure (such as the interfacial water layer) and the electric field. Considering the scientific and practical importance of hydrogen electrocatalytic reactions, delving into the regulation mechanisms of interface effects can not only aid in optimizing the design of hydrogen-related energy devices, but also help in understanding the intrinsic connections between interfacial structure and catalytic performance.

## MICROENVIRONMENTAL REGULATION FOR OXYGEN ELECTROCATALYSIS

Hydrogen and oxygen electrocatalysis form the foundation of water electrolyzers and hydrogen−oxygen fuel cells. Compared with hydrogen-related reactions, OER/ORR have much slower kinetics, and the high overpotential for the oxygen reaction side is the primary origin of device polarization. Therefore, optimizing the efficiency of oxygen electrocatalytic reactions is crucial for further improving device performance.

**Figure 4.**
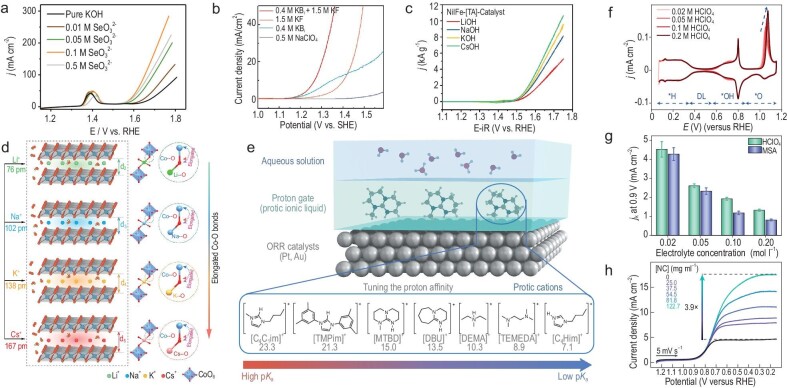
Microenvironmental regulation for oxygen electrocatalysis. (a) OER polarization curves of Ni(OH)_2_ in 1 M KOH with different concentrations of SeO_3_^2−^. Reproduced with permission from Ref. [14]. Copyright 2020 John Wiley & Sons, Inc. (b) OER polarization curves of Co(OH)_2_ in neutral electrolytes with different concentrations of borate and fluoride anions. Reproduced with permission from Ref. [16]. Copyright 2022 Elsevier Inc. (c) OER polarization curves of the NiFe-based catalysts recorded in alkaline electrolytes with different alkali metal cations. Reproduced with permission from Ref. [52]. Copyright 2022 John Wiley & Sons, Inc. (d) The schematic illustration of alkali cation intercalation and elongated Co–O bonds in CoOOH during the OER. Reproduced with permission from Ref. [53]. Copyright 2023 John Wiley & Sons, Inc. (e) The schematic illustration of tuning local proton activity for the ORR via protic cations of ionic liquids with different p*K*_a_. Reproduced with permission from Ref. [23]. Copyright 2021 Nature Publishing Group. (f) Cyclic voltammograms of Pt(111) in HClO_4_ solutions of different concentrations. (g) Summary of specific activities at 0.9 V (*vs*. RHE) of Pt(111) in HClO_4_ and methanesulfonic acid (MSA) of different concentrations. (f, g) Reproduced with permission from Ref. [55]. Copyright 2022 Nature Publishing Group. (h) ORR polarization curves of Pt/C in phosphate-buffered water (0.5 M KP_i_) solutions with different concentrations of zeolitic nanocrystals. Reproduced with permission from Ref. [57]. Copyright 2023 Nature Publishing Group.

Currently, it has been found that properly designed electrolyte compositions can accelerate OER, known as the electrolyte effect. Zhang *et al*. showcased that the addition of selenites (SeO_3_^2−^) or sulfate (SO_4_^2−^) in the electrolyte could significantly improve the OER performance of transition metal hydroxides such as Ni(OH)_2_, Cu(OH)_2_ and Co(OH)_2_ (Fig. 4a) [14]. *In situ* Raman spectroscopy and theoretical calculations indicate that these surface-adsorbed chalcogenates can significantly lower the Gibbs free energy of the intermediate of OER (*OOH), thus promoting OER activity. Similarly, surface-adsorbed carboxylate ligands (CLs) on bi/trimetallic layered double hydroxides (LDHs)/MOFs materials also exhibit a universal boosting effect for OER [15]. *In situ* spectroscopic evidence shows that the negatively charged CLs adsorbed on NiFeMn MOFs can act as mediators for proton transfer, accelerating and facilitating the adsorption, activation, and dissociation of OH^−^ to promote the OER. Besides these individual anions, the combinations of different anions have been found to exhibit interesting synergistic effects. Gong *et al*. discovered that the combination of borate and fluoride anions can have an intriguing synergistic effect for OER under neutral conditions, with the water oxidation current almost an order of magnitude higher than that for individual anions (Fig. 4b) [16]. This synergy was considered to be due to the hierarchical arrangement of these two anions at the interface, where borate anions in the inner Helmholtz layer promote proton-coupled electron transfer (PCET) steps, lowering the onset potential, while fluoride anions located further outside effectively disrupt the polymer hydrogen-bonding network in water, sustaining a larger current.

Similar to anions, alkali metal cations have also been found to significantly affect the OER performance of electrode materials, although the intrinsic role of the cations is still rather vague thus far. Koper *et al*. found that regardless of the presence of Fe impurities in alkaline electrolytes, the OER activities of NiOOH always show an intrinsic cation dependency [51]. *In situ* surface-enhanced Raman scattering (SERS) results suggest that the cation effect is related to the interaction with the superoxo OER intermediate (NiOO^−^). Cations with larger atomic numbers (such as Cs^+^) are supposed to be capable of better stabilizing the NiOO^−^−M^+^ species, thereby promoting O_2_ evolution. Advanced *in situ* characterization technologies provide new opportunities to probe the cations effect. Fischer *et al*. further demonstrated the correlation between the cations effect on the NiFe(OOH)-based electrocatalysts and the potential of maximum entropy (PME) using the laser-induced current transient (LICT) technique (Fig. 4c) [52]. The results suggest that Cs^+^ cations may accelerate the kinetics of the OER by altering the structure of the interfacial water layer. Combining the results from *operando* powder X-ray diffraction (PXRD), high-resolution transmission electron microscopy (HRTEM) and X-ray absorption spectroscopy (XAS), Luo *et al*. demonstrated that cations enter the layers of the Co-OOH catalyst through intercalation, leading to an increase in interlayer spacing (Fig. 4d) [53]. Cations with larger atomic numbers could facilitate the elongation of Co–O bonds, promoting the formation of high-valence OER-active *O–Co(IV) species. Co-OOH–Cs exhibits the lowest energy barrier for the rate-determining step (RDS), which explains the enhanced OER activity.

Similarly, the cations effects for ORR are also quite significant. Marković *et al*. proposed that hydrated alkali metal cations M^+^(H_2_O)*_x_* form OH_ad_–M^+^(H_2_O)*_x_* clusters at the interface via non-covalent interactions with OH_ad_ [18]. Cations with larger atomic numbers have lower hydration energies, leading to the higher ORR activities on Pt(111). Moreover, organic cations have been found capable of effectively promoting the ORR on Pt. Nakamura *et al*. studied the effect of tetraalkylammonium (TAA) cations with different alkyl chain lengths on the ORR on Pt(111) [54]. The high hydrophobicity of TAA cations with longer alkyl chains significantly alters the hydration structure of TAA. Among them, THA with the longest alkyl chain (*n* = 6) boosted the performance of Pt(111) by 8-fold. The high hydrophobicity of THA and its hydration shell weakens the binding of surrounding OH_ad_ and H_2_O_ad_ through weaker interactions, lowering the coverage of OH_ad_ and disrupting the stable hydrogen-bonding network, thus facilitating the entry of oxygen molecules onto the Pt(111) surface. Shao-Horn *et al*. recently reported the acceleration of the ORR kinetics of Au and Pt using protic cations of ionic liquids (Fig. 4e), where the ORR on Au mainly proceeds via the 2e^−^ pathway, whereas on Pt it follows the 4e^−^ pathway [23]. The ORR specific kinetic currents exhibit a volcano-shaped relationship *vs*. the p*K*_a_ of protic cations in the ionic liquids, with the maximum enhancement obtained when the p*K*_a_ values of the protic cations and the reaction intermediate in the RDS are similar. The optimal p*K*_a_ for Pt is around 15, close to the p*K*_a_ value of H_2_O, whereas the optimal p*K*_a_ for Au is around 11, close to the p*K*_a_ value of H_2_O_2_. *In situ* attenuated total reflection surface-enhanced infrared absorption spectroscopy (ATR-SEIRAS) and kinetic simulation results indicate a strong hydrogen bond interaction between the protic cations and the ORR intermediate, leading to faster proton tunneling kinetics and accelerated oxygen catalysis.

Earlier research suggested that the non-specifically adsorbed anions act as spectator species and have little impact on ORR. Yet recently, Koper *et al*. unearthed an unexpected effect of non-specifically adsorbed anions (ClO_4_^−^) on the ORR kinetics on Pt(111) (Fig. 4f, g) [55]. The role of the non-specifically adsorbed anions was explained by introducing the *O ↔ *OH transition as a new kinetic descriptor for ORR on Pt(111). This descriptor also successfully elucidated the impact of perfluorosulfonic acid (PFSA) ionomer and cations in alkaline media on the ORR activity on Pt(111). In addition, the mechanism for the significantly lower activity of M-N-C materials in acid (than in alkaline media) has also attracted attention. Chen *et al*. used FeCo-N6-C DAC as a model system and combined AIMD simulation with *in situ* ATR-SEIRAS to study the pH effect on its ORR activity [56]. The results indicate that the difference in activity stems from the distinct EDL structures in acidic and alkaline environments, where the orientation of interfacial water on electrode surfaces varies, impacting the formation of hydrogen bonds between the oxygenated intermediates and the interfacial water molecules, eventually leading to differences in ORR kinetics. In addition to the above ion effects, introducing microporous nanocrystals with hydrophobic internal surfaces and hydrophilic external surfaces into the electrolyte generates ‘microporous water’, which exhibits an O_2_-carrying capacity two orders of magnitude higher than pure water [57]. This enhancement in O_2_ supply at the electrode interface mitigates mass transport limitations (Fig. 4h), and thus facilitates the direct measurement of intrinsic ORR activity on Pt. This optimized mass transport strategy may offer a straightforward and effective intermediate testing method to bridge the gap between RDE and MEA measurement.

## MICROENVIRONMENTAL REGULATION FOR CO_2_ REDUCTION REACTION

**Figure 5.**
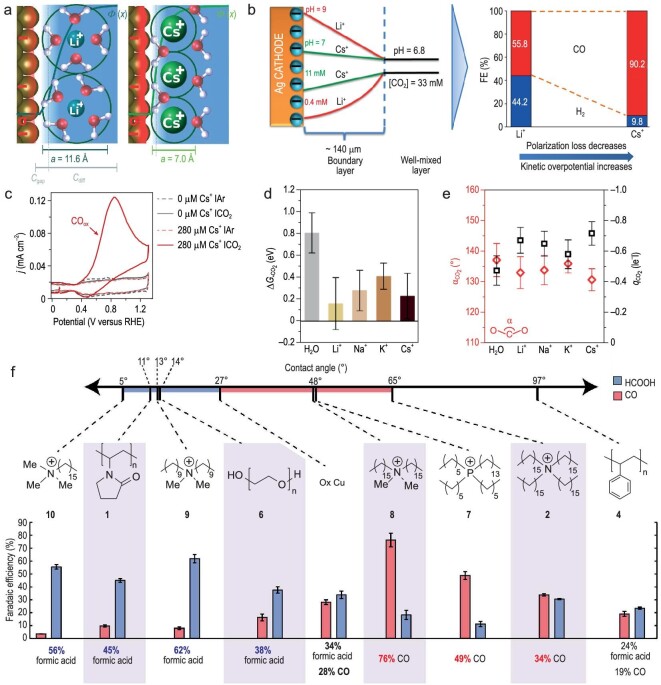
Microenvironmental regulation for CO_2_ reduction reaction. (a) Illustration of the origin of cation effects in field-driven electrocatalysis. Reproduced with permission from Ref. [61]. Copyright 2019 the Royal Society of Chemistry. (b) The schematic illustration of the distribution of pH and CO_2_ concentration in the boundary layer, and FE for CO increases and for H_2_ decreases with increasing cation size due to a decrease in polarization. Reproduced with permission from Ref. [58]. Copyright 2016 American Chemical Society. (c) The recorded anodic scans performed to oxidize any CO produced in argon and CO_2_ atmosphere. (d) Average CO_2_ adsorption Gibbs free energy at *U* = 0 V *vs*. standard hydrogen electrode (SHE) in the absence (grey) or presence (light to dark brown) of an alkali cation, with CO_2_ and the environment (Au–H_2_O or Au–H_2_O–M^+^) as the energy references. (e) *α*_CO2_ and CO_2_ Bader charge, *q*_CO2_, are shown in red and black, respectively. (c–e) Reproduced with permission from Ref. [64]. Copyright 2021 Nature Publishing Group. (f) Plotting the selectivity toward formic acid against the contact angle yields a marked relationship, with modifiers 2 and 4 as outliers at 65° and 97°, respectively. Reproduced with permission from Ref. [71]. Copyright 2019 American Chemical Society.

Electrochemical CO_2_RR powered by renewable energies is a promising route to transforming CO_2_ into value-added products and achieving sustainable carbon recycling. The microenvironment of electrocatalysis has a significant impact on CO_2_RR activity and selectivity. In comparison to HER and ORR, most CO_2_RR cases occur in a more negative potential range, where the cations tend to migrate to the cathode under the driving force of the electric field. These cations, serving as crucial components at the reaction interface, play a significant role in the CO_2_RR process. It has been noted in the majority of studies that the influence of alkali metal cations on CO_2_RR activity follows the order of Cs^+^ > K^+^ > Na^+^ > Li^+^ [58,59]. Although this order is consistent across various research works, the intrinsic mechanism of the cation effect remains controversial. Hori *et al*. proposed that cations could alter the OHP potential through specific adsorption, which in turn influences the concentration of H^+^ on the electrode surface, leading to different product selectivities [60]. On the other hand, Chan *et al*. refuted the possibility of cations undergoing specific adsorption (Fig. 5a) [61]; instead they suggested that cations would accumulate on the electrode surface through non-covalent interactions under CO_2_RR conditions, thus generating a high interfacial electric field. Weakly hydrated cations, with their smaller hydrated ionic radii, exhibit the least repulsion near the electrode. The resulting high concentration of cations leads to a higher surface charge density and a stronger interfacial electric field. Such modifications on the potential across the electron transfer plane could affect the apparent activation energy of the entire reaction, thereby influencing the adsorption and activation processes of CO_2_.

Distinct from the local electric field effect, Bell *et al*. proposed another theory, suggesting that cations could modulate the process by buffering the local pH (Fig. 5b) [58]. Due to electrolyte polarization during the CO_2_RR process, the local pH at the electrode surface tends to increase, and a large amount of OH^−^ would react with CO_2_ to form (bi)carbonate, thus lowering the accessibility of CO_2_. At the negatively charged cathode surface, the hydration shell of cations with larger atomic numbers experiences a stronger electrostatic field, thereby lowering its p*K*_a_ of hydrolysis. This lowered p*K*_a_ enables the hydrated cations to act as a buffering agent, suppressing the rise of local pH near the cathode. This leads to an increase in the local concentration of dissolved CO_2_, thereby facilitating the CO_2_RR process. Zhang *et al*. detected the changes in local pH during the CO_2_RR process on Au [62]. They found that cations with larger atomic numbers have a stronger buffering effect, with the interfacial pH values following the order of Li^+^ > Na^+^ > K^+^ > Cs^+^, which is in good agreement with Bell's results. Nørskov *et al*. suggested that the high local electric field produced by solvated cations can stabilize adsorbed intermediates with large dipole moments, thereby influencing the CO_2_RR process [63]. According to the GCS model, the strength of the electric field within the EDL under a given PZC is influenced by the size of the hydrated cations in the electrolyte. Cations with larger atomic numbers would experience stronger driving forces and accumulate near the electrode surface, generating a stronger interfacial electric field, which enhances the adsorption stability of some important CO_2_RR intermediates. Therefore, a higher CO_2_RR activity on copper was observed in the presence of cations with larger atomic numbers, which is attributed to their higher concentration in the OHP [59]. Koper *et al*. further refined this model and discovered that CO_2_RR does not occur in the absence of metal cations on gold, copper and silver electrodes (Fig. 5c) [64]. Only models considering the electrostatic interaction between metal cations and key intermediates can explain this observation. Through AIMD, the team further confirmed the stabilizing effect of partially desolvated metal cations on CO_2_ adsorption, activation, and the formation of CO_2_^−^ intermediates (Fig. 5d, e).

The anion effects differ from those for cations, with most studies focusing on adjusting the local pH (through distinct buffering capacities) and inducing catalyst restructuring. For instance, Hori *et al*. investigated the impact of anions with different buffering capacities (p*K*_a_: H_2_PO_4_^−^ < HCO_3_^−^ < ClO_4_^−^) on CO_2_RR [65]. They found that H_2_PO_4_^−^, having a strong buffering capacity, could easily neutralize OH^−^ near the copper electrode surface, maintaining a lower local pH, which correlates with high selectivity for H_2_ and CH_4_, while suppressing the formation of C_2_H_4_. In contrast, ClO_4_^−^, which lacks the buffering ability, led to a higher local pH at the copper electrode surface and enhanced the selectivity for C_2_H_4_. Cuenya *et al.* explored the influence of different halide anions on the restructuring process of metallic Cu surfaces [66]. Their findings indicated that different halide ions induce varying degrees of surface roughness. Compared with the original copper catalyst, the halide-ion–induced restructured catalysts exhibited improved selectivities for C_2+_ products.

The pH effect has been indirectly demonstrated through the effects of alkali metal cations and anions, significantly influencing reaction rate and product selectivity [58]. In CO_2_RR, the local pH near the electrode surface is typically higher than the pH of the bulk electrolyte, owing to the consumption of protons and the subsequent production of OH^−^ during surface reactions. Thus, the impact of local pH on CO_2_RR is more pronounced than that of bulk pH. For instance, Koper *et al.* discovered that as the CO_2_RR proceeds, the local pH increases, which promotes the HER on gold electrodes [67]. Controlling the local pH through mass transport conditions can regulate the rate of competing HER, thereby facilitating the CO_2_RR process on gold electrodes. In addition, CO_2_ as a reactant itself also acts as a buffering agent by reacting with OH^−^ at the electrode surface to form (bi)carbonates, thus significantly suppressing the rise in local pH during the CO_2_RR process. Therefore, the pH effect of CO_2_RR can be better observed in CORR with similar reaction pathways and RDS. For instance, the selectivity for acetate products in CORR can be significantly improved under conditions of high alkalinity. This may be due to the higher local pH facilitating the reaction of OH^−^ with certain intermediates to produce acetate [24]. To sum up, understanding the impact of local pH is of increasing importance for distinguishing between product selectivities.

Strongly alkaline microenvironments are generally believed to inhibit the HER and promote the formation rate of C_2+_ products on Cu electrodes, but this inevitably leads to a crossover between pH and cation effects. Sargent *et al*. suggested that OH^−^ on or near the copper surface can effectively lower the activation energy barriers for CO_2_RR and CO–CO coupling [68]. Experimental results showed that the onset of ethylene evolution at −0.165 V *vs.* RHE in 10 M KOH occurs almost simultaneously with the production of CO. This study proposed a plausible strategy to enhance the formation of C_2+_ products by increasing the pH of the electrolyte. However, this hypothesis contradicts the current understanding of the mechanisms involved in the formation of C_2+_ products. Recent research indicated that the RDS for C_2+_ product formation involves the dimerization of two adsorbed CO molecules through the Langmuir–Hinshelwood process, which does not involve proton transfer [69,70]. Thus, the formation rate of C_2+_ products theoretically should not depend so strongly on the pH of the electrolyte. Lu *et al.* discovered that at the same SHE potential, the concentration of Na^+^ has a greater promoting effect on the formation rate of C_2+_ products in CORR than does the concentration of OH^−^ [69]. This finding suggests that in strongly alkaline electrolytes, the primary factor enhancing the C_2+_ product formation rate could be the high concentration of cations, rather than OH^−^.

In addition to altering the composition of electrolytes to regulate cations, anions and pH, recent studies have shown that the modulation of reactant composition, catalyst surface functionalization (including modifications with organic molecules and polymers), and alterations in electrolysis methods can also regulate the interfacial microenvironment, thereby improving the activity and selectivity of the CO_2_RR [10,28,71]. Lu *et al*. demonstrated that the introduction of a moderate amount of O_2_ into the CO_2_RR system increased the production rate of oxygenates and hydrocarbons by 216 times [72]. Mechanistic studies revealed that the introduction of O_2_ created a hydroxyl-rich microenvironment on the surface of the Cu catalyst, thereby lowering the energy barriers for the formation of oxygenates and hydrocarbons. Bao *et al*. employed a co-feeding method of CO and CO_2_, whereby increasing the pressure of CO in the feeder resulted in a shift of the primary electrolysis product from ethylene to acetate [73]. Further studies revealed that this switching in selectivity is closely related to the increased coverage of CO intermediates and the rise in local pH. Specifically, ethylene is preferentially formed at low *CO coverage, whereas a high *CO coverage and elevated local pH would favor the formation of acetate. In terms of catalyst surface functionalization, Wang *et al*. innovatively developed a universal amino acid modification strategy, whereby copper electrodes functionalized with amino acids exhibited enhanced capability for hydrocarbon production regardless of electrode morphology [74]. Mechanistic studies indicated that the –NH_3_^+^ group of the adsorbed zwitterionic glycine could effectively facilitate the stable adsorption of the key intermediate CHO*. Bell *et al*. used bilayer cation- and anion-conducting ionomer coatings to control the catalytic microenvironment on the surface of copper electrodes [28]. Anion-exchange ionomers (AEIs), on account of their high CO_2_ solubility, increase the local ratio of CO_2_ to H_2_O. Cation-exchange ionomers (CEIs), on the other hand, raise the local pH by capturing the OH^−^ produced during the CO_2_RR and blocking the transport of buffering carbonate species into the catalyst microenvironment (via Donnan exclusion). In combination with pulsed electrolysis, the local ratio of CO_2_/H_2_O and pH were further increased on the ionomer-layer–modified Cu electrode, thereby improving the selectivity for C_2+_ products. Toma *et al.* employed a range of polymeric and molecular modifiers with different hydrophilicity and hydrophobicity to adjust the selectivity of CO_2_RR on Cu surfaces [71]. Their research revealed that protic species could enhance the selectivity for H_2_, hydrophilic additives could facilitate the formation of formic acid, and cationic hydrophobes would improve the selectivity for CO (Fig. 5f). The molecular dynamics simulations indicated that hydrophilic and hydrophobic modifiers would influence the formation of surface hydrides. Specifically, surface hydrides are more stable in the presence of hydrophobic species compared with hydrophilic ones. Consequently, the team suggested that the promotion of formic acid formation on hydrophilic surfaces is due to the weakening of the M–H bond. In contrast, hydrophobic species would lead to stronger M–H bonds, thus inhibiting the formation of formic acid and allowing the formation of CO to prevail. Pulsed electrolysis, by applying periodically alternating potentials, offers new possibilities for regulating the reaction microenvironment and the electrocatalytic process. For example, Cuenya *et al*. studied the effects of different anodic oxidation potentials (*E*_a_) during pulsing on CO_2_RR. They found that at *E*_a_ = 0.9 V *vs.* RHE, a significant number of grain boundaries and defect sites formed on the surface of copper particles, leading to a substantial increase in the Faradaic efficiency for C_2+_ products [75]. At *E*_a_ = 1.2 V *vs.* RHE, the regeneration of Cu_2_O consumed an excess of OH^−^, causing a decrease in the local pH at the catalyst surface, which favored the reaction pathway towards CH_4_ formation.

CO_2_RR could give a variety of products, which significantly adds to the complexity of its reaction pathways. The microenvironment not only affects the CO_2_RR kinetics, but also alters the selectivity of the final products. This provides a broader stage for microenvironment regulation strategies, but also increases the difficulty of understanding their intrinsic mechanisms.

## MICROENVIRONMENT REGULATION FOR PRACTICAL ELECTROCHEMICAL DEVICES

In practical electrochemical devices (often featuring high current densities), gases are often used directly (rather than in the dissolved form) as the reactants to ensure an efficient mass transport, which, on the other hand, also leads to a more complex reaction microenvironment. Consequently, investigations into microenvironments relevant to practical electrochemical devices are of great necessity. It is encouraging that some progress has been made in this field recently.

### Water electrolyzers and hydrogen–oxygen fuel cells

**Figure 6.**
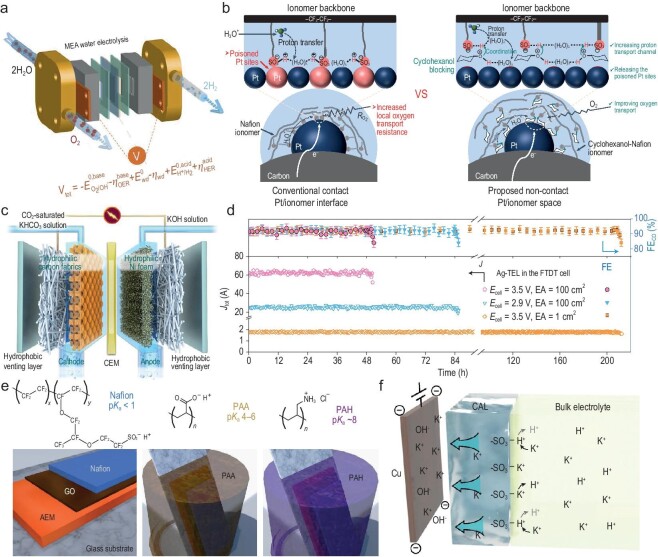
Microenvironmental regulation for practical electrochemical devices. (a) BPM water-dissociation measurements. WD is studied in an electrolyzer fed by only pure water. Reproduced with permission from Ref. [78]. Copyright 2020 American Association for the Advancement of Science. (b) Illustration of the conventional direct-contact Pt/ionomer interface and the proposed non-contact Pt/ionomer space for the cathode catalyst layer. Reproduced with permission from Ref. [25]. Copyright 2023 Nature Publishing Group. (c) Schematic illustration of FTDT cell. (d) Electrode stability tests for 1 cm^2^ and 100 cm^2^ FTDT cells at different cell voltages. (c, d) Reproduced with permission from Ref. [85]. Copyright 2022 Nature Publishing Group. (e) Molecular structures of the polyelectrolytes and schematic drawing of the membrane structure. Reproduced with permission from Ref. [86]. Copyright 2021 Nature Publishing Group. (f) Schematic illustration of ionic environment and transport near the catalyst surface functionalized by the PFSA ionomer. Reproduced with permission from Ref. [17]. Copyright 2021 American Association for the Advancement of Science.

Producing green hydrogen via water electrolysis is generally considered an ideal solution for achieving a future of renewable energy and carbon neutrality. Anion exchange membrane water electrolyzers (AEMWEs), benefiting from the use of inexpensive metal catalysts, offer notable advantages in cost. Typically, electrolyzing alkaline solutions may improve the performance and stability of AEMWEs in contrast with directly electrolyzing pure water, yet the exact mechanism underlying this performance enhancement remains uncertain. Weber *et al*. employed experimental and mathematical modeling approaches to expound the impact of hydroxide concentration on the performance of water electrolyzers [76]. They demonstrated the functional relationship between polarization curves and high-frequency resistance with hydroxide concentration. The findings indicated that increased hydroxide concentration is vital not only for the membrane and catalyst layer's ohmic resistance, but also for the reaction kinetics. Direct electrolysis of seawater is another cost-effective strategy, but it faces major challenges such as chloride corrosion and electrode passivation. Ling *et al*. introduced a Lewis acid layer (Cr_2_O_3_) on the surface of the catalyst (CoO*_x_*) to electrolyze water molecules and capture the *in situ* generated OH^−^ [77]. This *in situ* enrichment of OH^−^ effectively inhibits chloride corrosion on the catalyst surface and mitigates the issue of precipitate formation, achieving long-term stability of seawater electrolyzer exceeding 100 h at 500 mA cm^−2^. Compared with the same pH environments at both the cathode and anode in conventional electrolyzers, bipolar membrane (BPM) devices enable the independent selection of ideal pH conditions for each half-reaction, along with compatible catalysts. Boettcher *et al*. designed a BPM water electrolyzer that can provide an acidic environment for the HER side to accelerate its kinetics (Fig. 6a), while providing an alkaline environment on the OER side to allow for the utilization of low-cost base metal catalysts [78].

The performance and durability of fuel cells are influenced not only by the inherent properties of the catalysts but also by the microenvironment at the catalyst/ionomer interface. In proton exchange membrane fuel cells (PEMFCs), the adsorption of sulfonate groups in the ionomer on the surface of Pt-based catalysts would hamper their catalytic activities. To address this issue, Wei *et al*. employed cyclohexanol with the chair or boat conformations to coordinate with the ionomer, thus effectively blocking the way in which the ionomer is adsorbed onto the Pt surface (Fig. 6b) [25]. This approach led to the liberation of Pt active sites and significantly enhanced mass transport. On the other hand, the uniform distribution of ionomer can effectively improve oxygen mass transport at the Pt/ionomer interface, allowing for a lower Pt loading without incurring high voltage losses. Strasser *et al*. achieved this by introducing balanced *sp*^2^ pyridinic/pyrrolic/graphitic N functional groups interacting with the ionomer chains, ensuring homogeneous coverage of the ionomer on carbon supports [26]. Furthermore, considering the poor dispersion characteristics of conventional ionomers, Sung *et al*. employed supercritical isopropyl alcohol (IPA) in conjunction with water as cooperative polar co-solvents to boost the polarity of the solvent and intensify solvation by creating hydrogen bonds. This, in turn, led to the formation of an ionomer dispersion featuring colloidal particles significantly smaller on average than those present in commercially accessible dispersions [79]. For PEMFCs, water is solely formed at the cathode; in contrast, for AEMFCs, water is generated at the anode, while the water at the cathode is consumed. This poses more challenges for water management in the operation of AEMFCs. Mustain *et al*. incorporated hydrophobic components (PTFEs) into the gas diffusion layer and catalyst layer, resulting in gas diffusion electrodes (GDEs), and the water management was optimized by regulating the hydrophilicity/hydrophobicity of the catalyst/ionomer interface [80]. This design led to a stable operation, demonstrating continuous functioning for more than 1000 h at 600 mA cm^−2^.

### CO_2_RR electrolyzers

GDEs enhance the mass transport of CO_2_ to the active electrocatalysts, leading to greater current densities and carbon conversion efficiencies, thus constituting a key technology that is expected to achieve large-scale application of CO_2_RR. The GDE-based CO_2_RR electrolyzer could fail typically owing to carbonate deposition. Specifically, this is due to the high local concentration of carbonates formed from the reaction of CO_2_ with generated OH^−^, combined with the crossover of cations such as K^+^ and Na^+^ from the anolyte [81,82]. The carbonate deposition in GDEs would block the transport pathways of CO_2_, thus leading to device failure. Therefore, precise control of the microenvironment in GDEs, especially the regulation of carbonate chemistry, is crucial for CO_2_RR devices.

Janáky *et al*. developed an *operando* activation and regeneration process in which a solution containing alkaline metal cations is periodically injected into the cathode of a zero-gap electrolyzer to alleviate carbonate deposition [83]. This enables water-fed electrolyzers to operate with a CO_2_RR efficiency comparable to that using alkaline electrolytes (CO partial current density of 420 ± 50 mA cm^−2^, sustained for over 200 h). Additionally, the team demonstrated the scalability of this method across multiple electrolyzer stacks, each with an effective area of 100 cm^2^. Recently, Zhuang *et al.* employed a bifunctional ionomer as the polymer electrolyte to assemble an MEA electrolyzer capable of operating in pure water without adding any alkali metal cations, thereby avoiding the issue of carbonate deposition from the start [84]. When operated at a cell voltage of 3.54 V, ethylene is produced with an industrial-scale partial current density of 420 mA cm^−2^ without any electrolyte consumption. Chen *et al*. addressed the carbonate deposition issue by modifying the method of CO_2_ feeding [85]. They developed an electrolyzer that utilizes the forced convection of an aqueous CO_2_-saturated catholyte throughout a porous electrode (Fig. 6c). The decrease in local pressure led to increased exsolution of gaseous CO_2_ from dissolved CO_2_ and bicarbonate, which in turn significantly decreased the thickness of the diffusion layer (by a factor of ten). They assembled a scaled-up 4 × 100 cm^2^ electrolyzer stack that produced CO at a remarkable rate of 90.6 L h^−1^ and maintained good stability (Fig. 6d).

BPMs composed of a cation exchange layer (CEL) and an anion exchange layer (AEL) have also been explored for managing ion transport within the CO_2_RR electrolyzer [82]. A reverse-bias BPM can effectively control the ion transport across the bulk of the membrane. Here, cations from the anode are repelled by the AEL, while anions from the cathode are repelled by the CEL. This configuration effectively regulates the microenvironment at the GDL surface, suppressing the deposition of carbonates and the crossover of products. However, the acidic environment at CEL usually results in a low-pH microenvironment at the CEL/cathode interface, resulting in low CO_2_RR efficiency. In light of this, Mallouk *et al*. utilized ratiometric indicators and layer-by-layer polyelectrolyte assembly to deposit weakly acidic polyelectrolyte layers on the strongly acidic CEL (Fig. 6e) [86]. This arrangement allows for the measurement and manipulation of local pH at the GDL surface with a spatial resolution of tens of nanometers. The results indicate that the presence of the weak acid layer suppresses HER without adversely affecting CO_2_RR.

Compared with electrolyzers working under alkaline or neutral conditions, those working under acidic conditions typically exhibit higher carbon conversion efficiencies by inhibiting the formation of carbonates. However, the kinetically favored HER tends to dominate under acidic electrolytes. Sargent *et al*. addressed this challenge by modifying the catalyst surface with a cation-augmenting layer, maintaining a high concentration of K^+^ around the GDL surface (Fig. 6f) [17]. This approach enabled an efficient CO_2_RR process on Cu catalysts at pH <1 with a single-pass CO_2_ utilization of 77%. The research by Hu *et al*. further demonstrated that in acidic electrolytes, the hydrated alkali cations physisorbed on the cathode could alter the distribution of the electric field within the EDL [87]. This alteration inhibits the migration of hydronium ions towards the cathode, thereby impeding hydrogen evolution and facilitating the reduction of CO_2_. Moreover, Sinton *et al.* reported the development of a heterogeneous catalyst adlayer composed of covalent organic framework (COF) nanoparticles and cation exchange ionomers [27]. The imine and carbonyl-functionalized COFs formed uniformly distributed cation-carrying and hydrophilic–hydrophobic nanochannels. This structure led to a high local alkalinity and cation-enriched catalytic microenvironment near the GDL surface, which suppresses HER and enhances the conversion of CO_2_ to multi-carbon products on copper in strongly acidic conditions.

In summary, the research on the microenvironment effects at the EEI provides guidance for the design of practical electrochemical devices. However, it remains to be verified whether these anions, cations, and organic compounds would affect the long-term stability of ionomers or membranes. Therefore, we encourage the validation of strategies based on planar electrode systems in practical devices, with particular emphasis on long-term stability tests to drive the practicality of these devices.

## *IN SITU*/*OPERANDO* CHARACTERIZATION TECHNIQUES AND THEORETICAL SIMULATIONS

Conventional characterization techniques are often inadequate for observing the dynamic changes in mass/charge transport occurring at the EEI during electrocatalysis. To address this issue, a range of *in situ*/*operando* characterization techniques (including *in situ* visualization and spectroscopic methods) have been developed. Meanwhile, significant progress has been made in theoretical computational techniques based on EDL (AIMD) and electrochemical devices (continuum modeling) recently.

### *In situ* visualization methods

*In situ* visualization can help monitor the bubble behavior at the interface, which is crucial for guiding the design of more efficient devices, especially for water/CO_2_RR electrolyzers. Sun *et al*. developed a simple method called bubble pump consumption chronoamperometry (BPCC) involving the use of a gas supply unit and a data acquisition system (comprising high-speed camera, light source, and electrochemical workstation) [88]. By injecting individual oxygen bubbles into GDEs and monitoring the diffusion behavior along real-time current evolution, the exact correlation between the actual current over time and the dynamics of oxygen diffusion consumption was established. This enabled the determination of key parameters such as the gas diffusion coefficient and density of effective catalytic sites of GDEs. Zhang *et al*. observed the process of H_2_ evolution in proton exchange membrane electrolyzer cells (PEMECs) through a specialized high-speed microscopic visualization setup [89]. They demonstrated that gas evolution and bubble nucleation predominantly take place on catalyst layers at the edges of pores in the thin/tunable liquid/gas diffusion layers (TT-LGDLs). In addition to bubble behavior, *in situ* visualization can also help observe the wetting characteristics at the interface. Tileti *et al*. employed *in situ* liquid-phase TEM with an electrochemical approach to uncover the variation of wetting characteristics of cobalt-based oxide catalysts during potential cycling [90]. This method establishes the real-time correlations between the wetting behavior of materials and OER, providing visual evidence for the study of solid–liquid interfacial interactions in electrochemistry.

**Figure 7.**
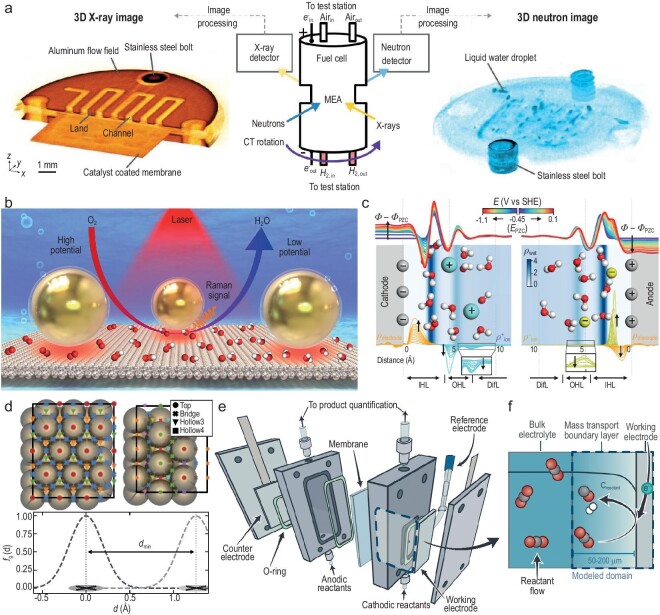
*In situ*/*operando* characterization techniques and theoretical simulations. (a) Fuel cell imaging schematic and sample neutron and X-ray images. Reproduced with permission from Ref. [91]. Copyright 2020 American Association for the Advancement of Science. (b) Model of shell-isolated nanoparticles (Au@SiO_2_ NPs, SHINs) at a Pt(111) surface and the mechanism of the ORR process revealed by the EC-SHINERS method. Reproduced with permission from Ref. [96]. Copyright 2019 Nature Publishing Group. (c) Representative structures of hydrated ions near the electrodes. Reproduced with permission from Ref. [102]. Copyright 2022 Nature Publishing Group. (d) Surface sites for the 3 × 4-(111) (top left) and 3 × 1-(211) (top right) slab models used for the site-partitioning of the AIMD trajectories. Symmetrically equivalent sites are shown in the same color. (Bottom) Schematic of Gaussian weights on surface sites with the Gaussian weighting function (*f_g_*) against the distance *d*. The weighting function overlap is depicted for two sites separated by the minimum distance *d*_min_. Reproduced with permission from Ref. [103]. Copyright 2020 AIP Publishing LLC. (e) Standard electrochemical compression cell and corresponding physical model schematic representation of a typical experimental setup, and (f) the corresponding modeled domain for the BL model. Reproduced with permission from Ref. [107]. Copyright 2023 Elsevier Inc.

The establishment of real-time characterization of component morphology and fluid dynamics is crucial for optimizing the performance and stability of fuel cells. Notably, Bazylak *et al*. combined advanced dual-modality tomography (simultaneous neutron and X-ray tomography) with sophisticated techniques for processing images (iterative reconstruction and metal artifact reduction) for characterization of fuel cell components (Fig. 7a) [91]. This method allows for the simultaneous, high-contrast, yet independent spatial and temporal analysis of both the morphology and moisture content of MEA components. In addition, Mustain *et al*. employed neutron imaging with *operando* micro–X-ray computed tomography (CT) to observe the distribution of liquid water over time and space within the functioning device [80]. This could lay the basis for optimizing water management.

### *In situ* spectroscopies

As a good complement to *in situ* visualization methods, *in situ* spectroscopies (including *in situ* X-ray scattering, surface-enhanced infrared/Raman spectroscopies) provide molecular-level experimental evidence for the chemical composition and adsorption–desorption information at the EEI.

*In situ* X-ray scattering can probe deep into condensed matter (such as solid phase and liquid phase) without hindering mass transport and electrochemical reactions, making it relatively easy to operate. The MEA components of CO_2_RR electrolyzers have long been struggling with poor stability attributed to cathode flooding and salt precipitation. However, the understanding of the mechanisms behind these local environmental changes remains rather limited. Seger *et al*. employed *operando* X-ray analysis combined with online gas chromatography and mass spectrometry to monitor electrolyte precipitation and salt deposition in the GDE of CO_2_RR electrolyzers [92]. Concurrently, direct evidence of bicarbonate salt precipitation at the cathode was identified, which exacerbates the issue of electrolyte flooding and shifts the reaction selectivity towards HER. Furthermore, the team employed *operando* wide-angle X-ray scattering (WAXS) to reveal how salt formation in the MEA cathode varies with changes in alkaline cations [93]. The findings indicate that salts with lower solubilities (e.g. Li/Na-based) would crystalize faster, which limits the CO_2_ from accessing the catalyst and enhances the competing HER. The key to avoiding salt precipitation is to use highly soluble alkali metal salts (e.g. CsHCO_3_) in the anolytes along with an optimal salt concentration ranging from 0.01 to 0.1 M.

Infrared/Raman spectroscopies serve as potent tools for molecular fingerprinting based on vibration modes. In previous sections discussing hydrogen/oxygen reactions and CO_2_RR, many works have demonstrated the importance of these two spectroscopies in analyzing microenvironmental effects [14,15,23,42,43,48,50]. Here, we will further elaborate on their respective characteristics, drawing from recent representative studies. Generally, IR spectroscopy detects dipole-moment-induced light absorption, whereas Raman spectroscopy assesses polarizability-induced inelastic scattering. Raman active centrosymmetric molecules are typically IR-inactive in IR owing to the mutual exclusion rule, and vice versa. Consequently, in the majority of instances, Raman and IR spectroscopies provide supplementary information to each other [94]. In order to address the enduring constraints of SERS related to structure and material applicability, Tian *et al.* introduced the shell-isolated nanoparticle-enhanced Raman spectroscopy (SHINERS) technique [95]. In SHINERS, the application of an ultra-thin and consistent silica shell on gold nanoparticles proves effective in amplifying the Raman signals from molecules near the nanoparticle surface without disruption. This enables Raman signal acquisition from various substrates. Li *et al*. investigated the ORR process at Pt(*hkl*) single-crystal surfaces using the SHINERS technique (Fig. 7b) [96]. They acquired direct spectral proof of *OH, *HO_2_ and O_2_^−^, elucidating the ORR mechanism at both the molecular and atomic levels under acidic and alkaline conditions. SHINERS’ distinct benefit lies in its specific adaptability to investigate the adsorption arrangement and catalytic mechanisms of probe molecules on surfaces of single crystals. The team further employed SHINERS to study the structure and dissociation procedure of interfacial water on Pd single-crystal surfaces, offering insights into the mechanism of structural arrangement of interfacial water and the influence of local cation disruption on the water structure [97].

ATR-SEIRAS is another commonly used *in situ* spectroscopic technique supplementary to SERS. Smith *et al*. investigated the pH gradients at the polycrystalline Cu and phosphate buffer interface during CO_2_RR using *in situ* SEIRAS [98]. The results indicate that at the potential of −1 V *vs.* RHE, where hydrocarbons are formed, there is a significant difference of 5 pH units between the local pH near the electrode surface and the bulk solution pH. This suggests that activity analysis under mass transport limitations must consider the impact of the large concentration gradients. Xu *et al*. employed ATR-SEIRAS to investigate the impact of different alkali metal cations on the Au electrode surface CO_2_ concentration [99]. At −0.8 V *vs*. RHE, the interfacial CO_2_ concentrations were found to be lowest in the presence of Cs^+^, providing a plausible explanation for the cation effect. Recently, Yang *et al*. combined *in situ* infrared nanospectroscopy (nano-FTIR) with *in situ* SERS and successfully revealed the formation pathway of NP/ordered-ligand interlayer induced by bias-induced dissociation of tetradecylphosphonic acid (TDPA) ligands on Ag NPs [100]. They also found that the accumulation of non-covalent interactions in the interfacial microenvironment during ligand dissociation promotes the development of surface-constrained electric fields, eventually enhancing the selectivity and activity for CO_2_RR. The combined spectroscopic approach provides an excellent example for investigating the dynamic response of microenvironments with nanoscale resolution, offering guidance for studying the interfacial microenvironments of nanomaterials.

### *Ab initio* simulation and continuum modeling

The progress of *in situ* characterization techniques has helped deepen the understanding of microenvironmental effects. However, these techniques still require further refinement to enhance the applicability in different EEIs and the reliability of data analysis. The theoretical simulation method can supplement, to some extent, the deficiency of experimental methods.

AIMD can efficiently establish the atomic-scale structure of the EDL, adjust the basic characteristics of the electrochemical interface, and deliberately introduce specific ions and solvents [101]. Kim *et al*. reproduced the experimentally observed characteristic peaks of electrochemical impedance spectroscopy (EIS) in dilute aqueous electrolyte through AIMD, and revealed that the origins for the two peaks observed at anodic and cathodic potentials are the electrosorption of ions and structural phase transition, respectively (Fig. 7c) [102]. This work demonstrates the advantages of the AIMD approach in building complex EDL structures, enabling direct validation of simulation results through comparison with experimental findings. In addition, AIMD can also predict solvent effects at solid/liquid interfaces with good accuracy. Chan *et al*. explored the solvation at charge-neutral metal/water interfaces using extensive AIMD simulations with commonly used continuum solvent models (Fig. 7d) [103]. They considered multiple metals (including Cu, Au, Pt) and various adsorbates relevant to electrochemical reactions (including CO_2_ reduction and oxygen redox), and reasonably quantified the solvation energies. These results provide a reference for establishing effective/parameterizable solvation models. Additionally, AIMD can effectively distinguish the configuration of the electrolyte at the interface and in the bulk liquid phase. Grob *et al*. analyzed the atomic configurations of water solvation layers on Pt(111) electrodes using AIMD [104]. The results revealed the difference of water molecule configurations between the surrounding solvation layer (through the oxygen atom bounding on Pt) and the bulk electrolyte (via H-up and H-down configurations), and less dense water solvation layers on hydrogen-covered Pt.

Benefiting from its advantages in simulating the EDL structure, AIMD is well suited as a powerful tool for analyzing microenvironment effects based on EEI. However, it is not as applicable for practical electrochemical devices working at high current densities because the corresponding microenvironment of porous electrodes often involves intricate multiphase fluid, mass transport, temperature, pressure, and other physical variables. Physics-based continuum modeling can play a crucial role in analyzing the microenvironment effects of electrochemical devices (include water electrolyzers, hydrogen–oxygen fuel cells, and CO_2_ electrolyzers), as it can link the device performance with electrode characteristics and operational conditions, and obtain theoretical product distributions and other device parameters [105]. This is crucial for the rational utilization of microenvironment effects to drive the design and preparation of high-performance devices. For instance, Weber *et al*. simulated the performance of Cu-based MEA for CO_2_RR through continuum modeling [106]. In this model, the team systematically investigated the effects of gas and liquid water transport, temperature, the properties of ionomer and membrane, heat transfer, and different feed conditions (liquid-fed and vapor-fed) on product distribution. The results indicate that higher temperatures (350 K) and sufficient water supply are advantageous for the formation of C_2+_ products. In addition, Bell *et al*. integrated continuum modeling and the covariance matrix adaptation evolutionary strategy (CMA-ES) to fit Tafel parameters for electrochemical CO_2_RR over Ag and Sn catalysts (Fig. 7e, f) [107]. This method eliminates subjective errors in fitting Tafel equations, establishing a more rigorous benchmark test. These results underscore the importance of accounting for mass transport and buffer kinetics in the analysis of the product of CO_2_ electrolyzers.

In summary, advanced *in situ*/*operando* techniques provide evidence of adsorption/desorption of reactants, products and, in particular, the intermediates, as well as real-time monitoring on the microenvironment status of device components. Further integration with the new theoretical calculation techniques can reasonably simulate the process of catalytic reactions and the operating status of devices. The development of these technologies and methods could well assist the study of microenvironment effects.

## CONCLUSION AND PERSPECTIVE

The past few decades have witnessed the booming development of sustainable electrocatalysis, covering both fundamental research in laboratories and commercializable applications in industry. The regulation of the microenvironment (by means of pH, cation/anion effects and constructing organic compound interface, for instance, caffeine) has proven successful in boosting the reaction kinetics and stability and improving the selectivity for specific target products in the hydrogen/oxygen reaction and carbon dioxide reduction reaction. The rapid advance of *in situ*/*operando* characterization techniques and theoretical simulation methods offers powerful tools to probe the catalytic pathways in further depth, which adds to understanding of EDL structure and microenvironment effect. Thanks to these advanced methodologies, scientists have begun to try unveiling the underlying mechanisms of microenvironment regulation, and found that the interfacial electric field, interfacial structures (such as water layer and hydrogen bond network) and the local concentration/coverage of relevant species (such as protons, hydroxide ion, reactants and intermediates) may play notable roles. On the basis of a more in-depth understanding on the electrode/electrolyte interface, the strategies for microenvironment regulation could readily translate into the design and fabrication of high-performance electrochemical devices for practical applications, including water/CO_2_ electrolyzers and fuel cells, promising a grand picture of large-scale implementation for green energy and sustainable development. Nevertheless, some related topics are still under debate, and a few subareas may need to be explored in further depth. At the end of this review, we share our views on several aspects that call for more thorough scrutiny.

- **Emphasis on a more accurate model for the electrical double layer model.** A clear and complete model for the EDL structure is the key to deciphering the microenvironment effect. It is of great necessity to conduct standardized measurements on the EDL properties (for instance, the electric fields for different EEIs, PZC) and to establish a more comprehensive database. The complexity in electrode preparation in metallic single-crystal electrode and the high requirements on the precision of electrocatalytic measurements call for advanced, high-efficiency characterization methodologies. In addition, the emerging *in situ*/*operando* characterization and theoretical simulation techniques are of great significance in providing more specific information on the EDL (such as the solvation of electrode surface, the location and interaction of solvated anions/cations and impact of the electric field).
- **Emphasis on the research on microenvironment regulation for low-cost catalysts based on non-platinum–group metals.** Historically, research on the microenvironment regulation of noble metal electrodes such as Pt electrodes helped to establish the correlation between EEI and hydrogen/oxygen reaction kinetics, yet the generality of this correlation for different electrode surfaces needs to be further validated. Currently, a vast number of low-cost, high-performance catalysts based on non-precious metals have been developed, and the research on microenvironment regulation for these electrode materials is of high necessity.
- **Emphasis on the research on microenvironment regulation of advanced electrochemical devices operating at industrially-relevant current densities.** For those advanced electrochemical devices such as MEA, the experimental tests are more complicated than those for planar electrodes, and the corresponding results are more subject to stochastic errors. Therefore, it is necessary to standardize the measurements for these advanced devices and to conduct multiple parallel tests so as to minimize errors. In addition, we noted that a few readily implementable ‘intermediate test techniques’ (between the conventional three-electrode configuration and the MEA) have emerged recently, such as promoting the mass transport for RDE [57], and using the floating electrode setup [108]. From a technoeconomic perspective, achieving high activity, selectivity, and stability at low overpotentials and at industrial-level current densities in advanced electrochemical devices presents greater challenges and significance. Another topic that needs particular attention is the effect of microenvironment on the long-term stability of devices. A typical example is the CO_2_ electrolyzer designed on the basis of cation effects, where the high-concentration cations could elevate catalytic efficiency and improve product selectivity, yet this could also exacerbate the problem of carbonate precipitation, thereby shortening the life cycle of the device. Therefore, in practice, a subtle balance needs to be maintained between the two conflicting aspects, so as to ensure the long-term operation of the devices featuring optimized reaction microenvironments.

## References

[1] Ross MB, De Luna P, Li Y et al. Designing materials for electrochemical carbon dioxide recycling. Nat Catal 2019; 2: 648–58. 10.1038/s41929-019-0306-7

[2] Jiao K, Xuan J, Du Q et al. Designing the next generation of proton-exchange membrane fuel cells. Nature 2021; 595: 361–9. 10.1038/s41586-021-03482-7 34262215

[3] Gao D, Arán-Ais RM, Jeon HS et al. Rational catalyst and electrolyte design for CO_2_ electroreduction towards multicarbon products. Nat Catal 2019; 2: 198–210. 10.1038/s41929-019-0235-5

[4] Lagadec MF, Grimaud A. Water electrolysers with closed and open electrochemical systems. Nat Mater 2020; 19: 1140–50. 10.1038/s41563-020-0788-3 33020614

[5] Yang Y, Peltier CR, Zeng R et al. Electrocatalysis in alkaline media and alkaline membrane-based energy technologies. Chem Rev 2022; 122: 6117–321. 10.1021/acs.chemrev.1c00331 35133808

[6] Wang G, Chen J, Ding Y et al. Electrocatalysis for CO_2_ conversion: from fundamentals to value-added products. Chem Soc Rev 2021; 50: 4993–5061. 10.1039/D0CS00071J 33625419

[7] Chen C, Jin H, Wang P et al. Local reaction environment in electrocatalysis. Chem Soc Rev 2024; 53: 2022–55. 10.1039/D3CS00669G 38204405

[8] Seh ZW, Kibsgaard J, Dickens CF et al. Combining theory and experiment in electrocatalysis: insights into materials design. Science 2017; 355: eaad4998. 10.1126/science.aad4998 28082532

[9] Gao Y, Liu B, Wang D. Microenvironment engineering of single/dual-atom catalysts for electrocatalytic application. Adv Mater 2023; 35: 2209654. 10.1002/adma.202209654 36813572

[10] Lv J-J, Yin R, Zhou L et al. Microenvironment engineering for the electrocatalytic CO_2_ reduction reaction. Angew Chem Int Ed 2022; 61: e202207252. 10.1002/anie.202207252 35819244

[11] Zheng W, Zhu R, Wu H et al. Tailoring bond microenvironments and reaction pathways of single-atom catalysts for efficient water electrolysis. Angew Chem Int Ed 2022; 61: e202208667. 10.1002/anie.202208667 35876718

[12] Zheng J, Sheng W, Zhuang Z et al. Universal dependence of hydrogen oxidation and evolution reaction activity of platinum-group metals on pH and hydrogen binding energy. Sci Adv 2016; 2: e1501602. 10.1126/sciadv.1501602 27034988 PMC4803484

[13] Govindarajan N, Xu A, Chan K. How pH affects electrochemical processes. Science 2022; 375: 379–80. 10.1126/science.abj2421 35084961

[14] Shi Y, Du W, Zhou W et al. Unveiling the promotion of surface-adsorbed chalcogenate on the electrocatalytic oxygen evolution reaction. Angew Chem Int Ed 2020; 59: 22470–4. 10.1002/anie.202011097 32897620

[15] Li C-F, Zhao J-W, Xie L-J et al. Surface-adsorbed carboxylate ligands on layered double hydroxides/metal–organic frameworks promote the electrocatalytic oxygen evolution reaction. Angew Chem Int Ed 2021; 60: 18129–37. 10.1002/anie.202104148 33982379

[16] Liu T, Chen Y, Hao Y et al. Hierarchical anions at the electrode-electrolyte interface for synergized neutral water oxidation. Chem 2022; 8: 2700–14. 10.1016/j.chempr.2022.06.012

[17] Huang JE, Li F, Ozden A et al. CO_2_ electrolysis to multicarbon products in strong acid. Science 2021; 372: 1074–8. 10.1126/science.abg6582 34083485

[18] Strmcnik D, Kodama K, van der Vliet D et al. The role of non-covalent interactions in electrocatalytic fuel-cell reactions on platinum. Nat Chem 2009; 1: 466–72. 10.1038/nchem.330 21378914

[19] Niu Z-Z, Gao F-Y, Zhang X-L et al. Hierarchical copper with inherent hydrophobicity mitigates electrode flooding for high-rate CO_2_ electroreduction to multicarbon products. J Am Chem Soc 2021; 143: 8011–21. 10.1021/jacs.1c01190 33913717

[20] Wakerley D, Lamaison S, Ozanam F et al. Bio-inspired hydrophobicity promotes CO_2_ reduction on a Cu surface. Nat Mater 2019; 18: 1222–7. 10.1038/s41563-019-0445-x 31384032

[21] Liang Y, Han Y, Li J-s *et al.* Wettability control in electrocatalyst: a mini review. J Energy Chem 2022; 70: 643–55. 10.1016/j.jechem.2021.09.005

[22] Sun Q, Oliveira NJ, Kwon S et al. Understanding hydrogen electrocatalysis by probing the hydrogen-bond network of water at the electrified Pt–solution interface. Nat Energy 2023; 8: 859–69. 10.1038/s41560-023-01302-y

[23] Wang T, Zhang Y, Huang B et al. Enhancing oxygen reduction electrocatalysis by tuning interfacial hydrogen bonds. Nat Catal 2021; 4: 753–62. 10.1038/s41929-021-00668-0

[24] Kim JYT, Sellers C, Hao S et al. Different distributions of multi-carbon products in CO_2_ and CO electroreduction under practical reaction conditions. Nat Catal 2023; 6: 1115–24. 10.1038/s41929-023-01082-4

[25] Chen F, Chen S, Wang A et al. Blocking the sulfonate group in Nafion to unlock platinum's activity in membrane electrode assemblies. Nat Catal 2023; 6: 392–401. 10.1038/s41929-023-00949-w

[26] Ott S, Orfanidi A, Schmies H et al. Ionomer distribution control in porous carbon-supported catalyst layers for high-power and low Pt-loaded proton exchange membrane fuel cells. Nat Mater 2020; 19: 77–85. 10.1038/s41563-019-0487-0 31570820

[27] Zhao Y, Hao L, Ozden A et al. Conversion of CO_2_ to multicarbon products in strong acid by controlling the catalyst microenvironment. Nat Synth 2023; 2: 403–12. 10.1038/s44160-022-00234-x

[28] Kim C, Bui JC, Luo X et al. Tailored catalyst microenvironments for CO_2_ electroreduction to multicarbon products on copper using bilayer ionomer coatings. Nat Energy 2021; 6: 1026–34. 10.1038/s41560-021-00920-8

[29] Fan J, Chen M, Zhao Z et al. Bridging the gap between highly active oxygen reduction reaction catalysts and effective catalyst layers for proton exchange membrane fuel cells. Nat Energy 2021; 6: 475–86. 10.1038/s41560-021-00824-7

[30] Lazaridis T, Stühmeier BM, Gasteiger HA et al. Capabilities and limitations of rotating disk electrodes versus membrane electrode assemblies in the investigation of electrocatalysts. Nat Catal 2022; 5: 363–73. 10.1038/s41929-022-00776-5

[31] Steinmann SN, Seh ZW. Understanding electrified interfaces. Nat Rev Mater 2021; 6: 289–91. 10.1038/s41578-021-00303-1

[32] Hamnett A . The electrode–electrolyte interface. Handbook of Fuel Cells. Hoboken: John Wiley & Sons. 2010.

[33] Gonella G, Backus EHG, Nagata Y et al. Water at charged interfaces. Nat Rev Chem 2021; 5: 466–85. 10.1038/s41570-021-00293-2 37118441

[34] Li C-Y, Le J-B, Wang Y-H et al. In situ probing electrified interfacial water structures at atomically flat surfaces. Nat Mater 2019; 18: 697–701. 10.1038/s41563-019-0356-x 31036960

[35] Favaro M, Jeong B, Ross PN et al. Unravelling the electrochemical double layer by direct probing of the solid/liquid interface. Nat Commun 2016; 7: 12695. 10.1038/ncomms12695 27576762 PMC5013669

[36] Ojha K, Arulmozhi N, Aranzales D et al. Double layer at the Pt(111)–aqueous electrolyte interface: potential of zero charge and anomalous Gouy–Chapman screening. Angew Chem Int Ed 2020; 59: 711–5. 10.1002/anie.201911929 PMC6973170 31682314

[37] Xu P, von Rueden AD, Schimmenti R et al. Optical method for quantifying the potential of zero charge at the platinum–water electrochemical interface. Nat Mater 2023; 22: 503–10. 10.1038/s41563-023-01474-8 36781952

[38] Rebollar L, Intikhab S, Oliveira NJ et al. “Beyond adsorption” descriptors in hydrogen electrocatalysis. ACS Catal 2020; 10: 14747–62. 10.1021/acscatal.0c03801

[39] Sheng W, Zhuang Z, Gao M et al. Correlating hydrogen oxidation and evolution activity on platinum at different pH with measured hydrogen binding energy. Nat Commun 2015; 6: 5848. 10.1038/ncomms6848 25569511

[40] Karlberg GS, Jaramillo TF, Skúlason E et al. Cyclic voltammograms for H on Pt(111) and Pt(100) from first principles. Phys Rev Lett 2007; 99: 126101. 10.1103/PhysRevLett.99.126101 17930522

[41] Chen X, McCrum IT, Schwarz KA et al. Co-adsorption of cations as the cause of the apparent pH dependence of hydrogen adsorption on a stepped platinum single-crystal electrode. Angew Chem Int Ed 2017; 56: 15025–9. 10.1002/anie.201709455 PMC5991472 28987066

[42] Yang X, Nash J, Oliveira N et al. Understanding the pH dependence of underpotential deposited hydrogen on platinum. Angew Chem Int Ed 2019; 58: 17718–23. 10.1002/anie.201909697 31571374

[43] Zhu S, Qin X, Yao Y et al. pH-dependent hydrogen and water binding energies on platinum surfaces as directly probed through surface-enhanced infrared absorption spectroscopy. J Am Chem Soc 2020; 142: 8748–54. 10.1021/jacs.0c01104 32306730

[44] Rebollar L, Intikhab S, Snyder JD et al. Kinetic isotope effects quantify pH-sensitive water dynamics at the Pt electrode interface. J Phys Chem Lett 2020; 11: 2308–13. 10.1021/acs.jpclett.0c00185 32125855

[45] Ryu J, Surendranath Y. Tracking electrical fields at the Pt/H_2_O interface during hydrogen catalysis. J Am Chem Soc 2019; 141: 15524–31. 10.1021/jacs.9b05148 31433173 PMC6777043

[46] Liu E, Li J, Jiao L et al. Unifying the hydrogen evolution and oxidation reactions kinetics in base by identifying the catalytic roles of hydroxyl-water-cation adducts. J Am Chem Soc 2019; 141: 3232–9. 10.1021/jacs.8b13228 30673227

[47] Shah AH, Zhang Z, Huang Z et al. The role of alkali metal cations and platinum-surface hydroxyl in the alkaline hydrogen evolution reaction. Nat Catal 2022; 5: 923–33. 10.1038/s41929-022-00851-x

[48] Li P, Jiang Y, Hu Y et al. Hydrogen bond network connectivity in the electric double layer dominates the kinetic pH effect in hydrogen electrocatalysis on Pt. Nat Catal 2022; 5: 900–11. 10.1038/s41929-022-00846-8

[49] Intikhab S, Rebollar L, Li Y et al. Caffeinated interfaces enhance alkaline hydrogen electrocatalysis. ACS Catal 2020; 10: 6798–802. 10.1021/acscatal.0c01635

[50] Zhao K, Chang X, Su H-S et al. Enhancing hydrogen oxidation and evolution kinetics by tuning the interfacial hydrogen-bonding environment on functionalized platinum surfaces. Angew Chem Int Ed 2022; 61: e202207197. 10.1002/anie.202207197 35941760

[51] Garcia AC, Touzalin T, Nieuwland C et al. Enhancement of oxygen evolution activity of nickel oxyhydroxide by electrolyte alkali cations. Angew Chem Int Ed 2019; 58: 12999–3003. 10.1002/anie.201905501 31250499

[52] Hou S, Xu L, Ding X et al. Dual in situ laser techniques underpin the role of cations in impacting electrocatalysts. Angew Chem Int Ed 2022; 61: e202201610. 10.1002/anie.202201610 PMC9321024 35274423

[53] Jia H, Yao N, Yu C et al. Unveiling the electrolyte cations dependent kinetics on CoOOH-catalyzed oxygen evolution reaction. Angew Chem Int Ed 2023; 62: e202313886. 10.1002/anie.202313886 37864480

[54] Kumeda T, Tajiri H, Sakata O et al. Effect of hydrophobic cations on the oxygen reduction reaction on single‒crystal platinum electrodes. Nat Commun 2018; 9: 4378. 10.1038/s41467-018-06917-4 30397202 PMC6218472

[55] Luo M, Koper MTM. A kinetic descriptor for the electrolyte effect on the oxygen reduction kinetics on Pt(111). Nat Catal 2022; 5: 615–23. 10.1038/s41929-022-00810-6

[56] Li P, Jiao Y, Ruan Y et al. Revealing the role of double-layer microenvironments in pH-dependent oxygen reduction activity over metal-nitrogen-carbon catalysts. Nat Commun 2023; 14: 6936. 10.1038/s41467-023-42749-7 37907596 PMC10618200

[57] Thorarinsdottir AE, Erdosy DP, Costentin C et al. Enhanced activity for the oxygen reduction reaction in microporous water. Nat Catal 2023; 6: 425–34. 10.1038/s41929-023-00958-9

[58] Singh MR, Kwon Y, Lum Y et al. Hydrolysis of electrolyte cations enhances the electrochemical reduction of CO_2_ over Ag and Cu. J Am Chem Soc 2016; 138: 13006–12. 10.1021/jacs.6b07612 27626299

[59] Resasco J, Chen LD, Clark E et al. Promoter effects of alkali metal cations on the electrochemical reduction of carbon dioxide. J Am Chem Soc 2017; 139: 11277–87. 10.1021/jacs.7b06765 28738673

[60] Murata A, Hori Y. Product selectivity affected by cationic species in electrochemical reduction of CO_2_ and CO at a Cu electrode. Bull Chem Soc Jpn 1991; 64: 123–7. 10.1246/bcsj.64.123

[61] Ringe S, Clark EL, Resasco J et al. Understanding cation effects in electrochemical CO_2_ reduction. Energ Environ Sci 2019; 12: 3001–14. 10.1039/C9EE01341E

[62] Zhang F, Co AC. Direct evidence of local pH change and the role of alkali cation during CO_2_ electroreduction in aqueous media. Angew Chem Int Ed 2020; 59: 1674–81. 10.1002/anie.201912637 31721382

[63] Chen LD, Urushihara M, Chan K et al. Electric field effects in electrochemical CO_2_ reduction. ACS Catal 2016; 6: 7133–9. 10.1021/acscatal.6b02299

[64] Monteiro MCO, Dattila F, Hagedoorn B et al. Absence of CO_2_ electroreduction on copper, gold and silver electrodes without metal cations in solution. Nat Catal 2021; 4: 654–62. 10.1038/s41929-021-00655-5

[65] Hori Y, Murata A, Takahashi R. Formation of hydrocarbons in the electrochemical reduction of carbon dioxide at a copper electrode in aqueous solution. J Chem Soc, Faraday Trans 1 1989; 85: 2309–26. 10.1039/f19898502309

[66] Gao D, Sinev I, Scholten F et al. Selective CO_2_ electroreduction to ethylene and multicarbon alcohols via electrolyte-driven nanostructuring. Angew Chem Int Ed 2019; 58: 17047–53. 10.1002/anie.201910155 PMC6899694 31476272

[67] Goyal A, Marcandalli G, Mints VA et al. Competition between CO_2_ reduction and hydrogen evolution on a gold electrode under well-defined mass transport conditions. J Am Chem Soc 2020; 142: 4154–61. 10.1021/jacs.9b10061 32041410 PMC7059182

[68] Dinh C-T, Burdyny T, Kibria MG et al. CO_2_ electroreduction to ethylene via hydroxide-mediated copper catalysis at an abrupt interface. Science 2018; 360: 783–7. 10.1126/science.aas9100 29773749

[69] Li J, Wu D, Malkani AS et al. Hydroxide is not a promoter of C_2+_ product formation in the electrochemical reduction of CO on copper. Angew Chem Int Ed 2020; 59: 4464–9. 10.1002/anie.201912412 31814246

[70] Li J, Chang X, Zhang H et al. Electrokinetic and in situ spectroscopic investigations of CO electrochemical reduction on copper. Nat Commun 2021; 12: 3264. 10.1038/s41467-021-23582-2 34075039 PMC8169934

[71] Buckley AK, Lee M, Cheng T et al. Electrocatalysis at organic–metal interfaces: identification of structure–reactivity relationships for CO_2_ reduction at modified Cu surfaces. J Am Chem Soc 2019; 141: 7355–64. 10.1021/jacs.8b13655 30929423

[72] He M, Li C, Zhang H et al. Oxygen induced promotion of electrochemical reduction of CO_2_ via co-electrolysis. Nat Commun 2020; 11: 3844. 10.1038/s41467-020-17690-8 32737312 PMC7395777

[73] Wei P, Gao D, Liu T et al. Coverage-driven selectivity switch from ethylene to acetate in high-rate CO_2_/CO electrolysis. Nat Nanotechnol 2023; 18: 299–306. 10.1038/s41565-022-01286-y 36635334

[74] Xie MS, Xia BY, Li Y et al. Amino acid modified copper electrodes for the enhanced selective electroreduction of carbon dioxide towards hydrocarbons. Energ Environ Sci 2016; 9: 1687–95. 10.1039/C5EE03694A

[75] Jeon HS, Timoshenko J, Rettenmaier C et al. Selectivity control of Cu nanocrystals in a gas-fed flow cell through CO_2_ pulsed electroreduction. J Am Chem Soc 2021; 143: 7578–87. 10.1021/jacs.1c03443 33956433 PMC8154520

[76] Liu J, Kang Z, Li D et al. Elucidating the role of hydroxide electrolyte on anion-exchange-membrane water electrolyzer performance. J Electrochem Soc 2021; 168: 054522. 10.1149/1945-7111/ac0019

[77] Guo J, Zheng Y, Hu Z et al. Direct seawater electrolysis by adjusting the local reaction environment of a catalyst. Nat Energy 2023; 8: 264–72. 10.1038/s41560-023-01195-x

[78] Oener SZ, Foster MJ, Boettcher SW. Accelerating water dissociation in bipolar membranes and for electrocatalysis. Science 2020; 369: 1099–103. 10.1126/science.aaz1487 32616669

[79] Ahn C-Y, Ahn J, Kang SY et al. Enhancement of service life of polymer electrolyte fuel cells through application of nanodispersed ionomer. Sci Adv 2020; 6: eaaw0870. 10.1126/sciadv.aaw0870 32064327 PMC6994205

[80] Peng X, Kulkarni D, Huang Y et al. Using operando techniques to understand and design high performance and stable alkaline membrane fuel cells. Nat Commun 2020; 11: 3561. 10.1038/s41467-020-17370-7 32678101 PMC7366663

[81] Wakerley D, Lamaison S, Wicks J et al. Gas diffusion electrodes, reactor designs and key metrics of low-temperature CO_2_ electrolysers. Nat Energy 2022; 7: 130–43. 10.1038/s41560-021-00973-9

[82] Ozden A, García de Arquer FP, Huang JE et al. Carbon-efficient carbon dioxide electrolysers. Nat Sustain 2022; 5: 563–73. 10.1038/s41893-022-00879-8

[83] Endrődi B, Samu A, Kecsenovity E et al. Operando cathode activation with alkali metal cations for high current density operation of water-fed zero-gap carbon dioxide electrolysers. Nat Energy 2021; 6: 439–48. 10.1038/s41560-021-00813-w 33898057 PMC7610664

[84] Li W, Yin Z, Gao Z et al. Bifunctional ionomers for efficient co-electrolysis of CO_2_ and pure water towards ethylene production at industrial-scale current densities. Nat Energy 2022; 7: 835–43. 10.1038/s41560-022-01092-9

[85] Wen G, Ren B, Wang X et al. Continuous CO_2_ electrolysis using a CO_2_ exsolution-induced flow cell. Nat Energy 2022; 7: 978–88. 10.1038/s41560-022-01130-6

[86] Yan Z, Hitt JL, Zeng Z et al. Improving the efficiency of CO_2_ electrolysis by using a bipolar membrane with a weak-acid cation exchange layer. Nat Chem 2021; 13: 33–40. 10.1038/s41557-020-00602-0 33288894

[87] Gu J, Liu S, Ni W et al. Modulating electric field distribution by alkali cations for CO_2_ electroreduction in strongly acidic medium. Nat Catal 2022; 5: 268–76. 10.1038/s41929-022-00761-y

[88] Li M, Xu W, Zhou D et al. Bubble pump consumption chronoamperometry for evaluating gas diffusion electrodes. Chem Catal 2023; 3: 100769. 10.1016/j.checat.2023.100769

[89] Kang Z, Yang G, Mo J et al. Novel thin/tunable gas diffusion electrodes with ultra-low catalyst loading for hydrogen evolution reactions in proton exchange membrane electrolyzer cells. Nano Energy 2018; 47: 434–41. 10.1016/j.nanoen.2018.03.015

[90] Shen T-H, Spillane L, Peng J et al. Switchable wetting of oxygen-evolving oxide catalysts. Nat Catal 2022; 5: 30–6. 10.1038/s41929-021-00723-w 35141468 PMC8799463

[91] Shrestha P, Lamanna JM, Fahy KF et al. Simultaneous multimaterial operando tomography of electrochemical devices. Sci Adv 2023; 9: eadg863. 10.1126/sciadv.adg8634 PMC10631724 37939178

[92] Moss AB, Garg S, Mirolo M et al. *In operando* investigations of oscillatory water and carbonate effects in MEA-based CO_2_ electrolysis devices. Joule 2023; 7: 350–65. 10.1016/j.joule.2023.01.013

[93] Garg S, Xu Q, Moss AB et al. How alkali cations affect salt precipitation and CO_2_ electrolysis performance in membrane electrode assembly electrolyzers. Energ Environ Sci 2023; 16: 1631–43. 10.1039/D2EE03725D

[94] Li J, Gong J. *Operando* characterization techniques for electrocatalysis. Energ Environ Sci 2020; 13: 3748–79. 10.1039/D0EE01706J

[95] Li JF, Huang YF, Ding Y et al. Shell-isolated nanoparticle-enhanced Raman spectroscopy. Nature 2010; 464: 392–5. 10.1038/nature08907 20237566

[96] Dong J-C, Zhang X-G, Briega-Martos V et al. In situ Raman spectroscopic evidence for oxygen reduction reaction intermediates at platinum single-crystal surfaces. Nat Energy 2019; 4: 60–7. 10.1038/s41560-018-0292-z

[97] Wang Y-H, Zheng S, Yang W-M et al. In situ Raman spectroscopy reveals the structure and dissociation of interfacial water. Nature 2021; 600: 81–5. 10.1038/s41586-021-04068-z 34853456

[98] Yang K, Kas R, Smith WA. In Situ infrared spectroscopy reveals persistent alkalinity near electrode surfaces during CO_2_ electroreduction. J Am Chem Soc 2019; 141: 15891–900. 10.1021/jacs.9b07000 31523949 PMC6788196

[99] Malkani AS, Anibal J, Xu B. Cation effect on interfacial CO_2_ concentration in the electrochemical CO_2_ reduction reaction. ACS Catal 2020; 10: 14871–6. 10.1021/acscatal.0c03553

[100] Shan Y, Zhao X, Fonseca Guzman M et al. Nanometre-resolved observation of electrochemical microenvironment formation at the nanoparticle–ligand interface. Nat Catal 2024; 7: 422–31. 10.1038/s41929-024-01119-2

[101] Li P, Jiao Y, Huang J et al. Electric double layer effects in electrocatalysis: insights from ab initio simulation and hierarchical continuum modeling. JACS Au 2023; 3: 2640–59. 10.1021/jacsau.3c00410 37885580 PMC10598835

[102] Shin S-J, Kim DH, Bae G et al. On the importance of the electric double layer structure in aqueous electrocatalysis. Nat Commun 2022; 13: 174. 10.1038/s41467-021-27909-x 35013347 PMC8748683

[103] Heenen HH, Gauthier JA, Kristoffersen HH et al. Solvation at metal/water interfaces: an *ab initio* molecular dynamics benchmark of common computational approaches. J Chem Phys 2020; 152: 144703. 10.1063/1.5144912 32295363

[104] Sakong S, Groß A. Water structures on a Pt(111) electrode from *ab initio* molecular dynamic simulations for a variety of electrochemical conditions. Phys Chem Chem Phys 2020; 22: 10431–7. 10.1039/C9CP06584A 31976502

[105] Bui JC, Lees EW, Pant LM et al. Continuum modeling of porous electrodes for electrochemical synthesis. Chem Rev 2022; 122: 11022–84. 10.1021/acs.chemrev.1c00901 35507321

[106] Weng L-C, Bell AT, Weber AZ. A systematic analysis of Cu-based membrane-electrode assemblies for CO_2_ reduction through multiphysics simulation. Energ Environ Sci 2020; 13: 3592–606. 10.1039/D0EE01604G

[107] Corpus KRM, Bui JC, Limaye AM et al. Coupling covariance matrix adaptation with continuum modeling for determination of kinetic parameters associated with electrochemical CO_2_ reduction. Joule 2023; 7: 1289–307. 10.1016/j.joule.2023.05.007

[108] Shi Z, Zhang X, Lin X et al. Phase-dependent growth of Pt on MoS_2_ for highly efficient H_2_ evolution. Nature 2023; 621: 300–5. 10.1038/s41586-023-06339-3 37704763

